# Attenuation of Oxidative Stress and Inflammatory Response by Chronic Cannabidiol Administration Is Associated with Improved n-6/n-3 PUFA Ratio in the White and Red Skeletal Muscle in a Rat Model of High-Fat Diet-Induced Obesity

**DOI:** 10.3390/nu13051603

**Published:** 2021-05-11

**Authors:** Patrycja Bielawiec, Ewa Harasim-Symbor, Klaudia Sztolsztener, Karolina Konstantynowicz-Nowicka, Adrian Chabowski

**Affiliations:** Department of Physiology, Medical University of Bialystok, Mickiewicz Str. 2C, 15-222 Bialystok, Poland; eharasim@umb.edu.pl (E.H.-S.); klaudia.sztolsztener@umb.edu.pl (K.S.); karolina.konstantynowicz@umb.edu.pl (K.K.-N.); adrian@umb.edu.pl (A.C.)

**Keywords:** cannabidiol, cannabis, inflammation, insulin resistance, lipids, oxidative stress

## Abstract

The consumption of fatty acids has increased drastically, exceeding the nutritional requirements of an individual and leading to numerous metabolic disorders. Recent data indicate a growing interest in using cannabidiol (CBD) as an agent with beneficial effects in the treatment of obesity. Therefore, our aim was to investigate the influence of chronic CBD administration on the n-6/n-3 polyunsaturated fatty acids (PUFAs) ratio in different lipid fractions, inflammatory pathway and oxidative stress parameters in the white and red gastrocnemius muscle. All the designed experiments were performed on Wistar rats fed a high-fat diet (HFD) or a standard rodent diet for seven weeks and subsequently injected with CBD (10 mg/kg once daily for two weeks) or its vehicle. Lipid content and oxidative stress parameters were assessed using gas–liquid chromatography (GLC), colorimetric and/or immunoenzymatic methods, respectively. The total expression of proteins of an inflammatory pathway was measured by Western blotting. Our results revealed that fatty acids (FAs) oversupply is associated with an increasing oxidative stress and inflammatory response, which results in an excessive accumulation of FAs, especially of n-6 PUFAs, in skeletal muscles. We showed that CBD significantly improved the n-6/n-3 PUFA ratio and shifted the equilibrium towards anti-inflammatory n-3 PUFAs, particularly in the red gastrocnemius muscle. Additionally, CBD prevented generation of lipid peroxidation products and attenuated inflammatory response in both types of skeletal muscle. In summary, the results mentioned above indicate that CBD presents potential therapeutic properties with respect to the treatment of obesity and related disturbances.

## 1. Introduction

Nowadays, according to the World Health Organization, obesity is one of the most significant health problems of the 21st century [1]. An increased prevalence of obesity in the world is attributed to many factors, including overnutrition, sedentary lifestyle, as well as many environmental and genetic factors, which were confirmed by a number of epidemiological and clinical studies [2]. During the progression of obesity, the excessive amounts of lipids are deposited in non-adipose tissues (e.g., liver, skeletal and cardiac muscle) leading to dyslipidemia, hyperglycemia and hyperinsulinemia [3,4,5]. These metabolic complications strongly correlate with the development of insulin resistance (IR), the occurrence of which plays a pivotal role in the pathogenesis of many chronic diseases such as type 2 diabetes mellitus (T2D), metabolic syndrome (MetS) and cardiovascular diseases (CVDs) [6,7]. 

**Scheme 1:**
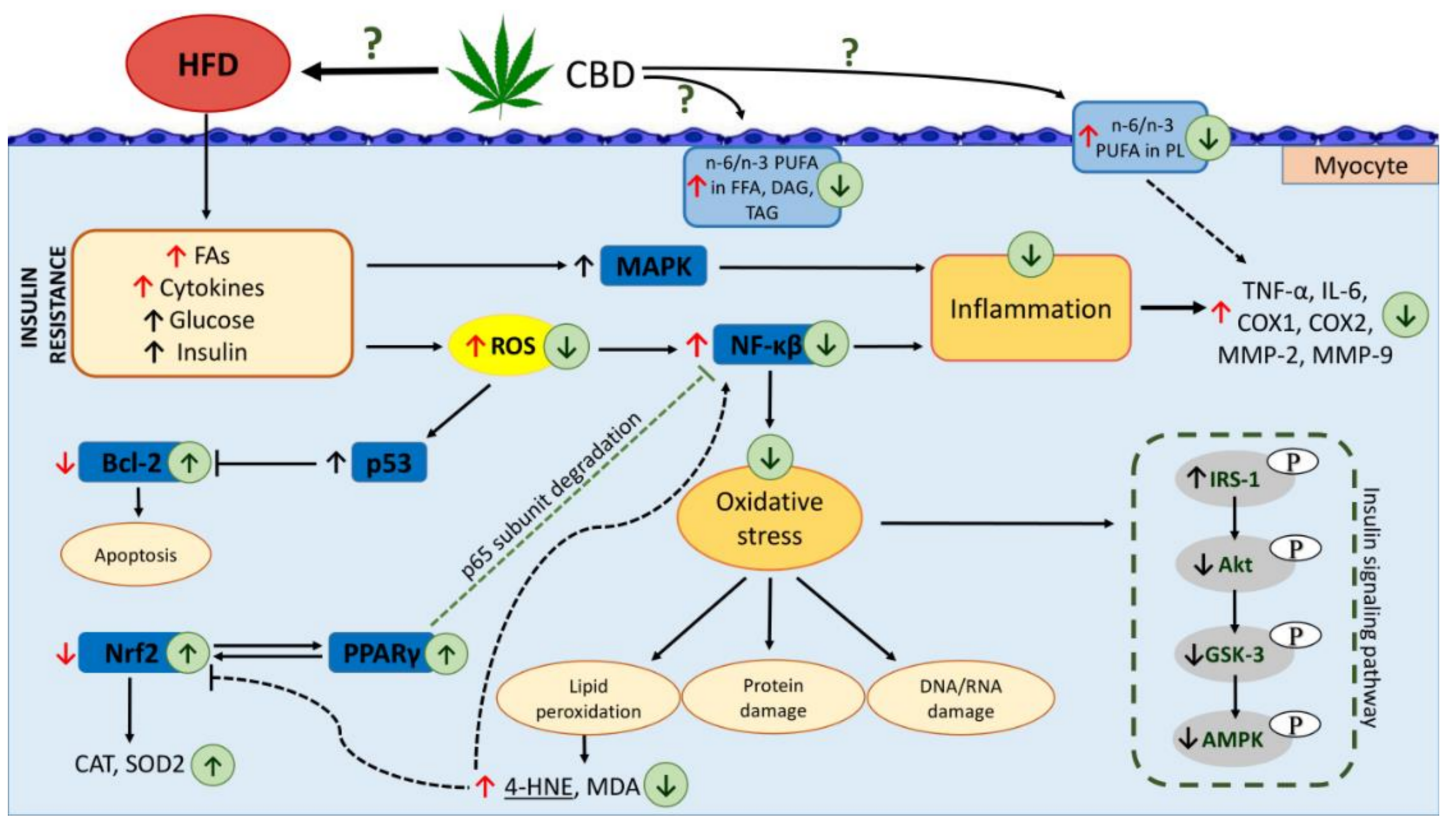
Effects of a high-fat diet (HFD) and two-week cannabidiol (CBD) administration on the n-6/n-3 polyunsaturated fatty acids (PUFAs) ratio in different lipid fractions (free fatty acids (FFAs), diacylglycerols (DAGs), triacylglycerols (TAGs) and phospholipids (PLs)), oxidative stress and inflammatory pathway in rat myocytes; ↑, increase; ↓, decrease; red arrow indicates the effects of seven weeks of high-fat diet feeding; black arrow indicates the effects of obesity; green arrow indicates the effects of two weeks of CBD treatment in high-fat diet fed rats; fatty acids (FAs); mitogen-activated protein kinase (MAPK); reactive oxygen species (ROS); p53 protein (p53); B cell lymphoma 2 (Bcl-2); nuclear factor κB (NF-κB); tumor necrosis factor α (TNF-α); interleukin 6 (IL-6); cyclooxygenase 1 (COX1); cyclooxygenase 2 (COX2); matrix metalloproteinase-2 (MMP-2), matrix metallo-proteinase-9 (MMP-9), peroxisome proliferator-activated receptor gamma (PPARγ); nuclear factor erythroid 2-related factor 2 (Nrf2); catalase (CAT), superoxide dismutase 2 (SOD2); 4-hydroxynonenal (4-HNE); malonyldialdehyde (MDA); insulin receptor substrate 1 (IRS-1), protein kinase B (Akt); glycogen synthase kinase 3 (GSK-3); 5’AMP-activated protein kinase (AMPK).

During this past decade, several studies reported the relevant role of chronic inflammation in the development of IR [8]. It was widely demonstrated that chronic overnutrition elicits an inflammatory response leading to systemic and tissue-specific low-grade inflammation through the release of proinflammatory cytokines, including interleukin 6 (IL-6), tumor necrosis factor alpha (TNF-α), as well as production of reactive oxygen species (ROS), which directly attenuate insulin signaling in insulin-sensitive tissues (Scheme 1) [8,9,10]. What is more, a high-fat diet (HFD) provides large amounts of polyunsaturated fatty acids (PUFAs), which are substrates for the synthesis of signaling molecules, eicosanoids (e.g., prostaglandins, prostacyclins, leukotrienes and thromboxanes) that affect functions of many tissues and organs in physiological and pathological conditions [11].

Moreover, data from numerous studies indicate that another mechanism which activates the proinflammatory cascade and appears to be of central importance is oxidative stress, which is defined as the imbalance between the production of ROS and reactive nitrogen species (RNS), and the effectiveness of enzymatic (e.g., catalase (CAT), superoxide dismutase (SOD) and glutathione peroxidase (GPx)) and non-enzymatic (e.g., reduced glutathione (GSH)) antioxidant protection [12]. Redox balance alterations in favor of ROS/RNS overproduction cause peroxidation of proteins and lipids as well as oxidative damage of nucleic acids, which may result in damage to cellular structures, including membranes, mitochondria and DNA (Scheme 1) [13].

In recent years, numerous studies based on newly emerging data have confirmed that the endocannabinoidome (eCBome) is a key player in the regulation of energy metabolism and its alterations. This complex lipid signaling system is also involved in the control of thermogenesis, neuromodulatory action and inflammatory processes [14,15]. By the turn of the last century, it was established that eCBome consists of endogenous ligands derived from long-chain PUFAs known as endocannabinoids (ECs), mostly *N*-arachidonoylethanolamine (anandamide (AEA)) and 2-arachidonoylglycerol (2-AG), with their own anabolic and catabolic pathways [16]. The action of ECs is mediated via widespread cannabinoid receptors CB_1_ and CB_2_, as well as many other molecular targets [17,18,19]. It is important to remark that ECs themselves have many structural congeners, and these compounds exist in dynamic balance with other lipid-derived mediators, including prostamides and eicosanoids [15]. Additionally, ECs serve as an endogenous source of arachidonic acid (AA) which is generated from AEA and 2-AG via the fatty acid amide hydrolase (FAAH) and monoacylglycerol lipase (MAGL), respectively [20].

Over the past century, the *Cannabis sativa* plant has been extensively studied for its medical properties. So far, more than 120 terpenophenolic compounds have been isolated from this medicinal plant [21]. Among all phytocannabinoids, cannabidiol (CBD) has been in the spotlight for several decades due to its excellent safety profile, lack of psychoactive effects and plenty of indicated therapeutic properties including neuroprotective, analgesic, anti-epileptic, anti-oxidative, anti-inflammatory and potential anti-obesity properties [22,23,24]. Considering “classic” cannabinoid CB_1_ and CB_2_ receptors, CBD has a very low affinity for these receptors, whereas it has been reported that this phytocannabinoid can modulate diverse G protein-coupled receptors (GPCRs) (e.g., GPR55 and GPR18), thermosensitive transient receptor potential (TRP) channels (vanilloid type-1 receptor, TRPV1) as well as opioid and peroxisome proliferator-activated receptors (PPARs: PPARα and PPARγ) [23,25,26]. Additionally, CBD has also been shown to alter the eCBome tone by inhibiting FAAH and subsequent AEA hydrolysis [27]. All the above mechanisms of CBD action demonstrate how complex the pharmacology it exhibits is, which must be taken into consideration in order to understand its therapeutic potential under various pathophysiological conditions. Taking this into consideration, the present study aimed to investigate the impact of CBD on the n-6/n-3 PUFA ratio in different lipid fractions (free fatty acids (FFAs), diacylglycerols (DAGs), triacylglycerols (TAGs) and phospholipids (PLs)), oxidative stress parameters as well as the inflammatory pathway in the white and red skeletal muscle (musculus gastrocnemius) of rats with obesity induced by a high-fat diet. Moreover, our research reveals a comprehensive relationship between the endocannabinoid system, the influence of n-6, n-3 PUFA contents and the oxidative stress along with inflammation associated with obesity. Furthermore, the study compares the CBD’s influence on two different metabolic types of skeletal muscle.

## 2. Materials and Methods

### 2.1. Animals and Experimental Protocol

The experiment was carried out on male Wistar rats weighing approximately 70–100 g obtained from the Center for Experimental Medicine of the Medical University of Bialystok, Poland. The animals were housed under standard holding conditions (22 ± 2 °C with a cycle of 12 h light/12 h dark) in plastic cages with unrestricted access to water and commercial pellet chow (Labofeed B, animal feed manufacturer “Morawski,” Kcynia, Poland). All the conducted procedures were evaluated and approved by the animal ethics committee in Olsztyn (No. 71/2018). The rats, after a period of acclimatization (seven days), were randomly divided into four groups: (1) control group fed a standard rodent diet (containing 12.4 kcal% fat, 57.1 kcal% carbohydrates and 30.5 kcal% protein), (2) CBD group fed a standard rodent diet and administered CBD, (3) HFD group fed a high-fat diet (containing 60 kcal% fat, 20 kcal% carbohydrates and 20 kcal% protein) and (4) HFD + CBD group fed a high-fat diet and administered CBD. Each experimental group consisted of ten rats. For seven weeks of the study, the animals received a standard diet or an HFD, and starting from the sixth week, rats were injected intraperitoneally (i.p.) with CBD or its vehicle. Respective control and HFD fed rats were injected i.p. once a day for two weeks with synthetic CBD (10 mg/kg, purity ≥ 99%; THC Pharm GmbH, Frankfurt, Germany) or its solvent (3:1:16, ethanol, Tween-80 and 0.9% NaCl). At the end of the experiment, twenty-four hours after the last dose of CBD or its vehicle, rats were anaesthetized i.p. with pentobarbital (80 mg/kg of body weight). Thereafter, whole blood was collected into test tubes with heparin through an inferior vena cava puncture and centrifuged to separate plasma. Muscle samples (red gastrocnemius muscle with predominant oxidative metabolism and white gastrocnemius muscle with largely anaerobic metabolism) were excised, and visible fatty tissue was mechanically removed. The obtained samples were at once frozen with aluminum tongs precooled in liquid nitrogen and then stored at −80 °C until final examination. Throughout the whole experiment, body weight of each rat was monitored (we observed significantly increased body weight in HFD fed rats; however, chronic CBD administration did not substantially affect the body weight in rats fed either a standard chow or a high-fat diet). Moreover, at the end of the experiment, we evaluated glucose and insulin concentrations as well as the HOMA-IR value, which was published previously [28].

### 2.2. Analysis of the Muscle and Plasma Lipid Contents

Intramuscular (FFA, DAG, TAG and PL) and plasma lipid (FFA and TAG) contents were determined by means of gas–liquid chromatography (GLC) as previously described [29]. In brief, the frozen muscle samples were pulverized with aluminum mortar precooled in liquid nitrogen. Subsequently, the muscle tissue and plasma lipids were extracted in a chloroform–methanol (2:1 vol/vol) solution using the Folch method [30]. Then, FFA, DAG, TAG and PL fractions were separated by thin-layer chromatography (TLC) on silica gel plates (Silica Plate 60, 0.25 mm; Merck, Darmstadt, Germany). Thereafter, the individual fatty acid methyl esters were quantified according to the standard retention times using GLC (Hewlett-Packard 5890 Series II gas chromatograph, HP-INNOWax capillary column). Total intramuscular FFA, DAG, TAG and PL as well as plasma FFA and TAG concentrations were estimated as the sum of the particular fatty acid species content in the selected fraction and expressed in nanomoles per gram of wet tissue and as nanomoles per milliliter in blood plasma.

### 2.3. Determination of Oxidative and Antioxidative Parameters

In order to determine the oxidative stress parameters, samples of the white and red skeletal muscles were homogenized in an ice-cold phosphate-buffered saline (PBS) with the addition of protease and phosphatase inhibitors (Roche Diagnostics GmbH, Manheim, Germany) at 20 mg of tissue/1 mL PBS for catalase (CAT), superoxide dismutase 2 (SOD2) and total antioxidant capacity (TAC); at 10 mg of tissue/90 μL PBS for advanced glycation end product (AGE) and 4-hydroxynonenal (4-HNE); in a radioimmunoprecipitation assay (RIPA) buffer containing a cocktail of protease and phosphatase inhibitors (Roche Diagnostics GmbH, Manheim, Germany) at 25 mg of tissue/250 μL RIPA for malondialdehyde (MDA) determination. Subsequently, the homogenates in the PBS were centrifuged for five minutes at 12,000× *g* at 4 °C to assess CAT, SOD2 and TAC, and for evaluation of AGE and 4-HNE, they were centrifuged at 5000× *g* for five minutes at 4 °C. For MDA determination, homogenates in the RIPA buffer were centrifuged for 10 min at 1600× *g* (4 °C). Thereafter, the supernatants were collected and stored in aliquots at −80 °C for later use.

The concentrations of CAT and SOD2 in muscle homogenates were measured with the use of a commercial ELISA kit from Cloud-Clone Corp. (Houston, TX, USA) following the manufacturer’s instructions. The intensity of colored products was determined at the 450-nm wavelength in a hybrid multimode microplate reader (Synergy H1TM, BioTek Instruments, Winooski, VT, USA) and, for each measurement, the calculated values were based on the obtained standard curve. The CAT and SOD2 concentrations were expressed in nanograms and picograms per milligram of tissue, respectively.

In order to evaluate the TAC parameter in muscle samples, we used a colorimetric method (TAC assay kit, Abcam, Cambridge, UK). The absorbance was measured colorimetrically at 570 nm (Synergy H1TM, BioTek Instruments, Winooski, VT, USA). The value of the TAC parameter was calculated according to the manufacturer’s instructions and expressed in nanomoles per milligram of tissue.

To determine intramuscular MDA content, we used a commercial kit purchased from Cayman Chemical Company (Ann Arbor, MI, USA) using the thiobarbituric acid reactive substances (TBARS) method. The intensity of reaction products (MDA–TBA adducts) was measured colorimetrically at the 530-nm wavelength in a hybrid multimode microplate reader (Synergy H1TM, BioTek Instruments, Winooski, VT, USA). MDA concentration was calculated based on the standard curve and expressed as nanomoles per milligram of tissue.

Intramuscular AGE and 4-HNE concentrations were assessed using ELISA kits from Biorbyt (Cambridge, UK) according to the manufacturer’s protocol. The absorbance was measured spectrophotometrically at the 450-nm wavelength in a hybrid multimode microplate reader (Synergy H1TM, BioTek Instruments, Winooski, VT, USA). The values of AGE and 4-HNE were calculated from standard curves and expressed in nanograms and picograms per milligram of tissue, respectively.

### 2.4. Western Blotting

Routine Western blotting procedure was used to determine total protein expression as it was reported in detail previously [31,32]. Briefly, red and white muscle samples were homogenized in an ice-cold RIPA buffer with the addition of protease and phosphatase inhibitors (Roche Diagnostics GmbH, Manheim, Germany). The determination of the total protein concentration in muscle homogenates was performed using the bicinchoninic acid (BCA) protein assay method with bovine serum albumin (BSA) as a standard. Subsequently, homogenates (30 μg of proteins) were reconstituted in the Laemmli buffer and separated on CriterionTM TGX Stain-Free precast gels (Bio-Rad, Hercules, CA, USA). Then, the proteins were transferred to nitrocellulose or polyvinylidene fluoride (PVDF) membranes in wet and semi-dry conditions, respectively. Next, the membranes were blocked in the Tris-buffered saline with Tween-20 (TBST) and 5% non-fat dry milk or 5% BSA and then incubated overnight at 4 °C with primary antibodies, i.e., cyclooxygenase-1 (COX-1, 1:500; Abcam, Cambridge, UK), cyclooxygenase-2 (COX-2, 1:500; Santa Cruz Biotechnology, Inc., Dallas, TX, USA), 5-lipoxygenase (5-LO, 1:1500; Abcam, Cambridge, UK), 15-lipoxygenase (15-LO, 1:500; Santa Cruz Biotechnology, Inc., Dallas, TX, USA), peroxisome proliferator-activated receptor alpha (PPARγ, 1:500; Santa Cruz Biotechnology, Inc., Dallas, TX, USA), nuclear factor-ĸB (NF-ĸB, 1:500; Cell Signaling Technology Inc., Danvers, MA, USA), tumor necrosis factor α (TNF-α, 1:500; Santa Cruz Biotechnology, Inc., Dallas, TX, USA), interleukin 6 (IL-6, 1:3000; Abcam, Cambridge, UK), nuclear factor erythroid 2-related factor 2 (Nrf-2, 1:500; Abcam, Cambridge, UK), B cell lymphoma 2 (Bcl-2, 1:500; Cell Signaling Technology Inc., Danvers, MA, USA), matrix metalloproteinase-2 (MMP-2, 1:2500; Abcam, Cambridge, UK) and matrix metalloproteinase-9 (MMP-9, 1:5000; Abcam, Cambridge, UK). Thereafter, the membranes were incubated with horseradish peroxidase (HRP) and conjugated secondary antibodies (1:3000, Santa Cruz Inc., Dallas, TX, USA). In order to visualize protein bands, the chemiluminescence substrate (Clarity Western ECL Substrate; Bio-Rad, Hercules, CA, USA) was used, and then the obtained signals were quantified densitometrically with the use of the ChemiDoc visualization system (Image Laboratory Software Version 6.0.1; Bio-Rad, Warsaw, Poland). The expression of selected proteins was quantified with stain-free gels and the total protein normalization method (Bio-Rad, Hercules, CA, USA) (see Supplementary File S1). All the data are demonstrated as the percentage of the control group based on six independent determinations.

### 2.5. Statistical Analysis

The experimental data are expressed as mean values ± SD or percentage of the control group based on six independent determinations. The obtained results were subjected to the Shapiro–Wilk test and the Bartlett’s test to assess the distribution of values and homogeneity of the variance. Statistical differences between groups were assessed by one-way test ANOVA followed by the appropriate post-hoc test with the use of GraphPad Prism version 7.0 for Windows (GraphPad Software, La Jolla, CA, USA). The results were considered to be statistically significant at *p* < 0.05.

## 3. Results

### 3.1. Effect of Two-Week CBD Treatment on the n-6 and n-3 PUFA Ratio in the White and Red Skeletal Muscles As Well As Plasma of Rats Subjected to Standard and High-Fat Diets

Induction of obesity by feeding rats a HFD resulted in a significant reduction in the pool of the n-6 PUFAs, i.e., of linoleic acid (LA) (−16.9%, *p* < 0.05), arachidonic acid (AA) (−10.1%, *p* < 0.05), and also of n-3 PUFAs, i.e., of α-linolenic acid (ALA) (−16.0%, *p* < 0.05) and eicosapentaenoic acid (EPA) (−17.5%, *p* < 0.05) in the FFA fraction (Table 1), in the white gastrocnemius muscle compared with the controls. We only observed an increase in the content of docosahexaenoic acid (DHA) (+32.9%, *p* < 0.05; Table 1) under the same conditions. Concomitantly, the rats fed both the standard and the HFD after the introduction of CBD exhibited a substantially elevated content of LA (+42.6% and +62.5%, *p* < 0.05, vs. control group, respectively; +95.6%, *p* < 0.05, vs. the HFD group; Table 1), AA (+52.8% and +173.7%, *p* < 0.05, vs. the control group, respectively; +204.5%, *p* < 0.05, vs. the HFD group; Table 1), ALA (+29.9%, *p* < 0.05, vs. the control group; +30.7%, *p* < 0.05, vs. the HFD group; Table 1) and DHA (+52.8% and +161.1%, *p* < 0.05, vs. the control group, respectively; +96.5%, *p* < 0.05, vs. the HFD group; Table 1) in the white skeletal muscle. However, compared to the control conditions, rats from the HFD group exhibited a considerably decreased EPA content after two-week CBD administration (−29.0%, *p* < 0.05; Table 1) in the same muscle type. In contrast, in the red gastrocnemius muscle, we noticed a significant increment in the fatty acid content of the FFA fraction, i.e., of LA (+76.0%), AA (+41.7%), ALA (+27.7%) and DHA (+75.1%) (*p* < 0.05; Table 2), in the rats subjected to a high-fat diet in comparison with the control rats, with the exception of EPA (*p* > 0.05; Table 2). Interestingly, the total intramuscular content of FFA fraction fatty acids in the rats fed either the standard or the high-fat diet was considerably greater in the chronic presence of CBD: LA (+39.2% and +63.5%, *p* < 0.05, vs. the control group, respectively; Table 1), AA (+41.7% and +99.3%, *p* < 0.05, vs. the control group, respectively; +40.6%, *p* < 0.05, vs. the HFD group; Table 2), ALA (33.7% and 22.0%, *p* < 0.05, vs. the control group, respectively; Table 2), EPA (+33.6% and +20.1%, *p* < 0.05, vs. the control group, respectively; +22.0%, *p* < 0.05, vs. the HFD group; Table 2) and DHA (+31.3% and +102.8%, *p* < 0.05, vs. the control group, respectively; +15.8%, *p* < 0.05, vs. the HFD group; Table 2) in the oxidative muscle. 

Concomitantly, in the DAG fraction, we observed that high-fat diet feeding caused a significant decrease of n-6 PUFAs (LA (−22.2%) and AA (−19.9%)) and n-3 PUFAs (ALA (−22.0%)), whereas the DHA content was markedly elevated (+42.0%) in the white gastrocnemius muscle in comparison with the control group (*p* < 0.05; Table 3). Interestingly, CBD treatment in the rats fed a standard chow resulted in a substantially reduced content of LA (−14.6%, *p* < 0.05, vs. the control group; Table 2) and increased AA (+32.5%, *p* < 0.05, vs. the control group; Table 3) in the white skeletal muscle. However, in the same muscle type, the HFD group after CBD administration was characterized by a significant increment in the pool of LA (+26.8%, *p* < 0.05, vs. the HFD group; Table 3), AA (+130.4%, *p* < 0.05, vs. the control group; +187.5%, *p* < 0.05, vs. the HFD group; Table 3) and DHA (+107.9%, *p* < 0.05, vs. the control group; +46.4%, *p* < 0.05, vs. the HFD group; Table 3), whereas the content of ALA and EPA was considerably reduced (−24.5% and −23.0%, respectively) in comparison with the controls (*p* < 0.05; Table 3). Conversely, in the red gastrocnemius muscle, changes in the total fatty acid content of the DAG fraction in the rats subjected to a high-fat diet were accompanied by a significant increase in the content of n-6 PUFAs (LA (+82.9%) and AA (+18.5%)) as well as all of n-3 PUFAs (ALA (+31.3%), EPA (+23.1%) and DHA (+93.6%)) in comparison with the rats fed a standard diet (*p* < 0.05; Table 4). CBD treatment in the rats fed the standard chow markedly enhanced the AA and EPA content (+29.9% and +39.7%, *p* < 0.05, vs. the control group, respectively; Table 4) with no change in LA, ALA and DHA levels (*p* > 0.05; Table 4) in the oxidative muscle. Similarly, we observed that the HFD-fed group after CBD administration showed a significantly greater accumulation of LA (+70%, *p* < 0.05, vs. the control group; Table 4), AA (+78.8%, *p* < 0.05, vs. the control group; +50.9%, *p* < 0.05, vs. the HFD group; Table 4), ALA (+44.8%, *p* < 0.05, vs. the control group; +10.2%, *p* < 0.05, vs. the HFD group; Table 4), EPA (+80.4%, *p* < 0.05, vs. the control group; +46.5%, *p* < 0.05, vs. the HFD group; Table 4) and DHA (+131.3%, *p* < 0.05, vs. the control group; +19.4%, *p* < 0.05, vs. the HFD group; Table 4) in the pool of the DAG fraction in the red gastrocnemius muscle. 

As shown in Table 5, the rats from the HFD group had an increased content of LA (+337.1%), AA (+64.8%), ALA (+138.1%) and DHA (+88.1%) compared to the control group in the white skeletal muscle’s TAG fraction (*p* < 0.05; Table 5) with no alterations in the EPA levels (*p* > 0.05; Table 5). Moreover, we noticed that two-week CBD treatment in the rats fed the standard chow considerably elevated only DHA levels (+50.0%, *p* < 0.05, vs. the control group; Table 5) of the TAG fraction in the white skeletal muscle. Concomitantly, in the same muscle type, we observed that CBD administration to rats after the HFD course substantially enhanced the levels of n-6 PUFAs, i.e., of LA and AA (+187.0% and +72.5%, *p* < 0.05, vs. the control group, respectively; Table 5) in the TAG fraction, whereas the LA content in the same experimental group compared to the corresponding untreated HFD group was decreased (−34.4%, *p* < 0.05; Table 5). Furthermore, we noticed a similar effect of two-week CBD treatment in the case of n-3 PUFAs such as ALA (+95.4%, *p* < 0.05, vs. the control group; Table 5) and DHA (+171.9%, *p* < 0.05, vs. the control group; +43.9%, *p* < 0.05, vs. the HFD group; Table 5), although only the EPA content was significantly reduced (−37.5%, *p* < 0.05, vs. the control group; Table 5) in the white gastrocnemius muscle. With respect to the red skeletal muscle, high-fat feeding considerably intensified the accumulation of LA and DHA (+116.9% and +161.6%, respectively; *p* < 0.05; Table 6) in the TAG fraction in comparison with the rats fed the standard diet. Interestingly, compared to the control conditions, we did not observe any significant alterations in the PUFA composition of the TAG fraction in the CBD group in the same muscle type (*p* > 0.05; Table 6). However, the HFD group after CBD administration exhibited a substantially reduced level of LA (−37.7%, *p* < 0.05, vs. the HFD group; Table 6) along with a substantial rise in n-3 PUFAs EPA (+56.2%, *p* < 0.05, vs. the HFD group; Table 6) and DHA (+249.9%, *p* < 0.05, vs. the control group; +33.7%, *p* < 0.05, vs. the HFD group; Table 6). 

Our study demonstrated that the total fatty acid composition of PL was altered by the high-fat diet in both white and red gastrocnemius muscles. Regarding the glycolytic muscle, we noticed a pronounced decrease in the content of LA, ALA and EPA in the rats subjected to the HFD (−39.5%, −43.9% and −46.8%, respectively; *p* < 0.05; Table 7) compared to the control rats, whereas only the DHA content was increased (+12.7%, *p* < 0.05; Table 7) in the same conditions. CBD given to the rats fed a standard chow did not affect in a statistically significant manner the PUFA content in the white skeletal muscle in the PL fraction. However, when CBD was administered to rats after the HFD course, it considerably reduced the content of LA, ALA and EPA (−27.2%, −37.0% and −37.3%, respectively; *p* < 0.05; Table 7) and simultaneously increased the AA and DHA levels (+21.6% and +69.2%, respectively; *p* < 0.05; Table 7) in comparison with the control conditions. In the same experimental rats, compared to the HFD group alone, we observed a substantially increased accumulation of all PUFAs: LA, AA, ALA, EPA and DHA in the PL fraction (+20.3%, +19.9%, +12.3%, +17.7% and +50.1%, respectively; *p* < 0.05; Table 7). Concomitantly, in the PL fraction of the red gastrocnemius muscle, the HFD-fed rats were characterized by considerably elevated AA and DHA content (+21.5% and +25.6%, respectively; *p* < 0.05; Table 8) with a parallel decline in the levels of ALA and EPA (−42.4% and −30.1%, respectively; *p* < 0.05; Table 8) along with no changes in the LA content (*p* > 0.05; Table 8) compared to the controls. CBD administration while feeding rats with the standard chow resulted in a pronounced increase in n-6 PUFAs, that is, LA (+21.0%) and AA (+19.4%) as well as in n-3 PUFAs, that is, ALA (+11.1%) and DHA (+53.3%) (*p* < 0.05; Table 8) in comparison with the control group. Additionally, we observed a significant increase in the PUFA content of the PL fraction after prolonged CBD administration in the high-fat diet rats: LA (+27.2%, *p* < 0.05, vs. the control group; +23.1%, *p* < 0.05, vs. the HFD group), AA (+53.4%, *p* < 0.05, vs. the control group; +26.3%, *p* < 0.05, vs. the HFD group) and DHA (+78.7%, *p* < 0.05, vs. the control group; +42.2%, *p* < 0.05, vs. the HFD group) in the red skeletal muscle (Table 8). These CBD effects were accompanied by a reduction in the ALA and EPA content (−32.7% and −25.9%, respectively; *p* < 0.05; Table 8) compared to the control rats in the red skeletal muscle.

In the plasma FFA fraction of the rats fed an HFD, we noticed significantly increased levels of n-6 PUFAs, i.e., of LA (+68.5%) and AA (+107.3%) and of n-3 PUFA DHA (+31.8%) (*p* < 0.05; Table 9). Interestingly, CBD administration to the animals fed a standard diet caused a pronounced elevation of all the PUFAs: LA, AA, ALA, EPA and DHA (+52.3%, +88.2%, +43.6%, +35.0% and +108.1%, respectively; *p* < 0.05; Table 9) in comparison with the corresponding control rats. Similarly, the high fat diet-fed rats after CBD administration presented a significantly elevated level of LA (+167.9%, *p* < 0.05, vs. the control group; +59.0%, *p* < 0.05, vs. the HFD group), AA (+90.5%, *p* < 0.05, vs. the control group), ALA (+95.5%, *p* < 0.05, vs. the control group; +67.2%, *p* < 0.05, vs. the HFD group) and DHA (81.2%, *p* < 0.05, vs. the control group; +37.5%, *p* < 0.05, vs. the HFD group). The only fatty acid of plasma FFA PUFAs with the content reduced in the rats fed an HFD after two-week CBD treatment was EPA (−23.6%, *p* < 0.05, vs. the control group; Table 9). However, in comparison with the corresponding untreated HFD group, the EPA content was substantially enhanced (+33.5%, *p* < 0.05; Table 9). On the other hand, in the plasma TAG fraction, the high-fat diet resulted in a considerable decrease in fatty acids belonging to n-3 PUFAs: ALA (−46.6%), EPA (−65.8%) and DHA (−56.1%) (*p* < 0.05, vs. the control group; Table 10). Moreover, we observed a marked elevation of the DHA content in the plasma TAG fraction of animals fed a standard diet after prolonged CBD administration (+22.9%, *p* < 0.05; Table 10) compared to the controls. Consistently, CBD given to the rats after the HFD course significantly reduced the EPA and DHA content (−61.5% and −47.4%, *p* < 0.05, vs. the control group; Table 10). However, in comparison with the HFD group alone, we reported in the same experimental group a substantially declined content of AA in the chronic presence of CBD (−18.4%, *p* < 0.05; Table 10) and, conversely, a significantly greater content of ALA (+60.0%, *p* < 0.05; Table 10). 

**Figure 1:**
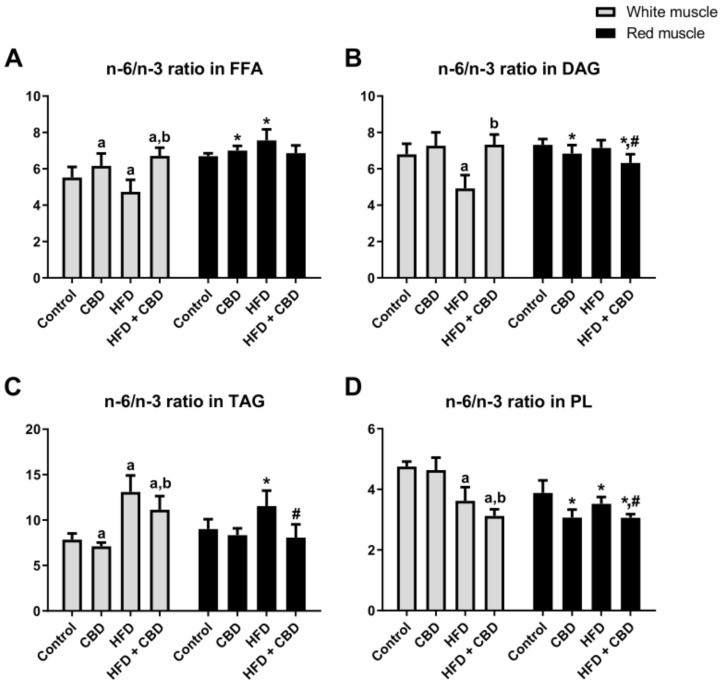
Intramuscular n-6/n-3 PUFA ratio in the (**A**) free fatty acid (FFA), (**B**) diacylglycerol (DAG), (**C**) triacylglycerol (TAG) and (**D**) phospholipid (PL) fractions in the white and red gastrocnemius muscles in the control (standard diet) and high-fat diet (HFD) groups after two-week cannabidiol (CBD) treatment. The data are expressed as mean values ± SD, *n* = 10 in each group. ^a^ *p* < 0.05 indicates a significant difference: the control group vs. the examined group in the white gastrocnemius muscle; ^b^ *p* < 0.05 indicates a significant difference: HFD vs. HFD + CBD in the white gastrocnemius muscle. * *p* < 0.05 indicates a significant difference: the control group vs. the examined group in the red gastrocnemius muscle; ^#^ *p* < 0.05 indicates a significant difference: HFD vs. HFD + CBD in the red gastrocnemius muscle.

Our study demonstrated that the high-fat diet substantially reduced the n-6/n-3 PUFA ratio in the FFA and DAG fractions (−14.2% and −27.6%, *p* < 0.05; Figure 1A,B, respectively) in the white gastrocnemius muscle compared to the controls, whereas CBD administration considerably enhanced it (+41.7% and +49%.0%, *p* < 0.05; Figure 1A,B, respectively) in comparison with the HFD group alone. Regarding the FFA fraction, we also found that two-week CBD treatment significantly increased the n-6/n-3 PUFA ratio in the rats fed either the standard chow or the high-fat diet in the white gastrocnemius muscle (+11.6% and +21.6%, *p* < 0.05; Figure 1A) in comparison with the control group. On the other hand, in the red gastrocnemius muscle, we observed elevated n-6/n-3 PUFA ratio in the rats fed HFD in the FFA fraction (+12.9%, *p* < 0.05, vs. the control group; Figure 1A), whereas chronic CBD treatment resulted in a significant decrease in the same experimental group (−9.3%, *p* < 0.05, vs. the HFD group; Figure 1A). Interestingly, in the red gastrocnemius muscle, we observed a substantial reduction in the n-6/n-3 PUFA ratio in the rats subjected to standard chow or high-fat diet feeding after CBD administration in the DAG (−6.6% and −13.7%, *p* < 0.05, vs. the control group, respectively; −11.6%, *p* < 0.05, vs. the HFD group; Figure 1B) and PL fractions (−21.0% and −21.2%, *p* < 0.05, vs. the control group, respectively; −13.1%, *p* < 0.05, vs. the HFD group; Figure 1D). With respect to the TAG fraction (Figure 1C) in the white and red gastrocnemius muscles, we observed that the HFD-fed groups exhibited a significantly increased n-6/n-3 PUFA ratio (+66.8% and +28.1%, respectively; *p* < 0.05; Figure 1C) in comparison with the control group. Concomitantly, two-week CBD treatment caused a substantial reduction in the n-6/n-3 PUFA ratio (−14.9% and −30.1%, respectively; *p* < 0.05; Figure 1C) compared to the HFD group alone. Moreover, the n-6/n-3 PUFA ratio in the TAG fraction only in the white gastrocnemius muscle was considerably reduced by CBD administration in the rats fed a standard chow (−9.5%, *p* < 0.05; Figure 1C) and, conversely, in the rats fed the HFD, it was significantly elevated (+42.0%, *p* < 0.05; Figure 1C) in comparison with the control group. Simultaneously, in the PL fraction, we observed that the HFD-fed groups exhibited a markedly decreased n-6/n-3 PUFA ratio in both the white (−23.8%, *p* < 0.05; Figure 1D) and the red (−9.3%, *p* < 0.05; Figure 1D) gastrocnemius muscle compared to the control conditions. Moreover, CBD administration to animals being on an HFD in the white gastrocnemius muscle resulted in a pronounced reduction of the n-6/n-3 PUFA ratio in the PL fraction (−13.9%, *p* < 0.05, vs. the HFD group; Figure 1D). 

**Figure 2:**
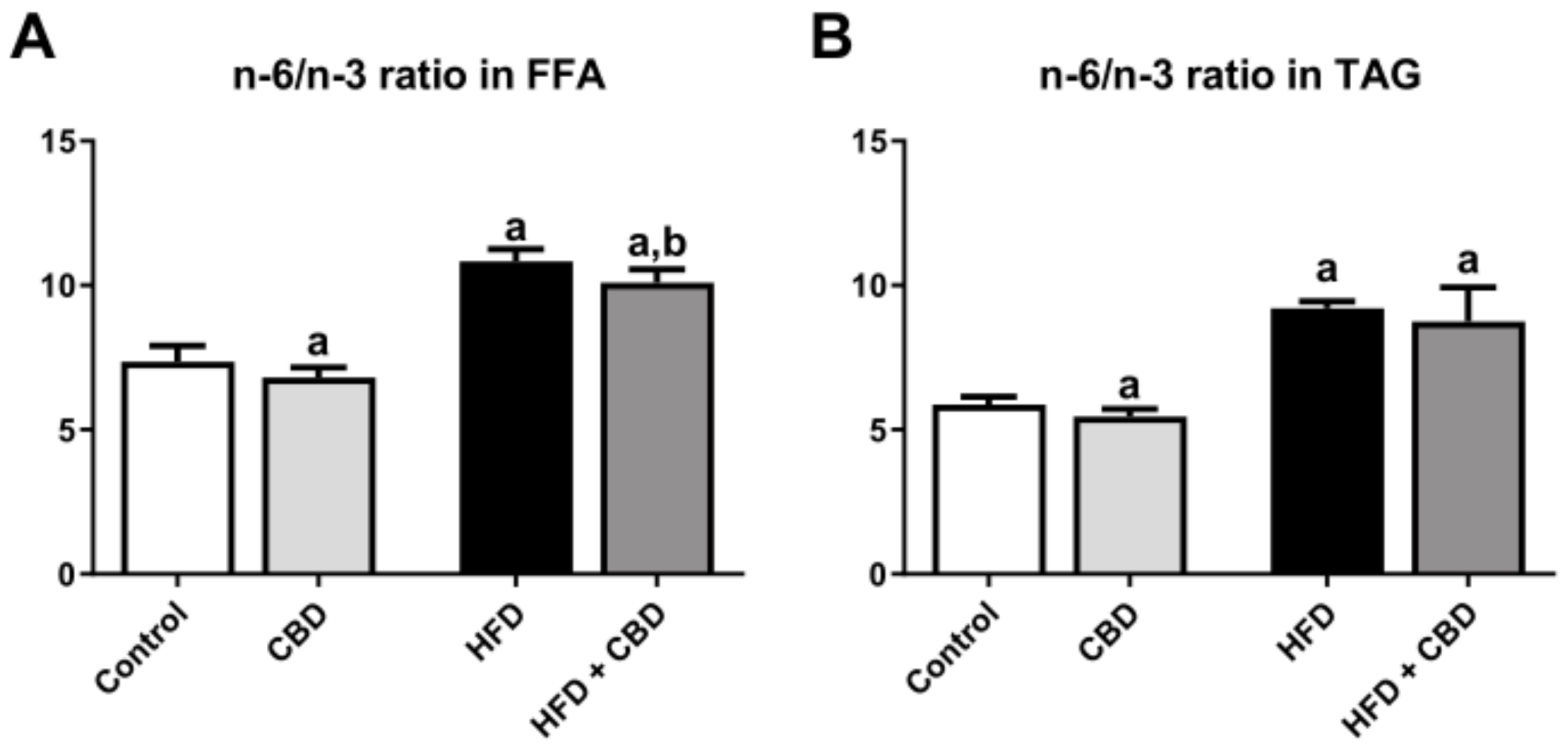
Plasma n-6/n-3 PUFA ratio in the (**A**) free fatty acid (FFA) and (**B**) triacylglycerol (TAG) fractions in the control (standard diet) and high-fat diet (HFD) groups after two-week cannabidiol (CBD) treatment. The data are expressed as mean values ± SD, *n* = 10 in each group. ^a^ *p* < 0.05 indicates a significant difference: the control group vs. the examined group; ^b^ *p* < 0.05 indicates a significant difference: HFD vs. HFD + CBD.

Induction of obesity by high-fat diet feeding resulted in a significantly increased n-6/n-3 PUFA ratio in both HFD-fed groups (untreated and treated with CBD) in the plasma FFA (+47.3% and +37.3%, respectively; *p* < 0.05; Figure 2A) and TAG fractions (+57.1% and +49.1%, respectively, *p* < 0.05; Figure 2B) compared to the control rats. Furthermore, in the plasma of the rats fed a standard diet and injected with CBD, we observed a markedly decreased n-6/n-3 PUFA ratio in the FFA (−7.4%, *p* < 0.05; Figure 2A) and TAG (−6.9%, *p* < 0.05; Figure 2B) fractions in comparison with the control group. Importantly, the n-6/n-3 PUFA ratio was substantially decreased in the chronic presence of CBD during the high-fat diet feeding course in the plasma FFA fraction (−6.8%, *p* < 0.05, vs. the HFD group; Figure 2A). 

### 3.2. Effect of Two-Week CBD Treatment on the Oxidative and Antioxidative Parameters in the White and Red Skeletal Muscles of Rats Subjected to Standard and High-Fat Diets

**Figure 3:**
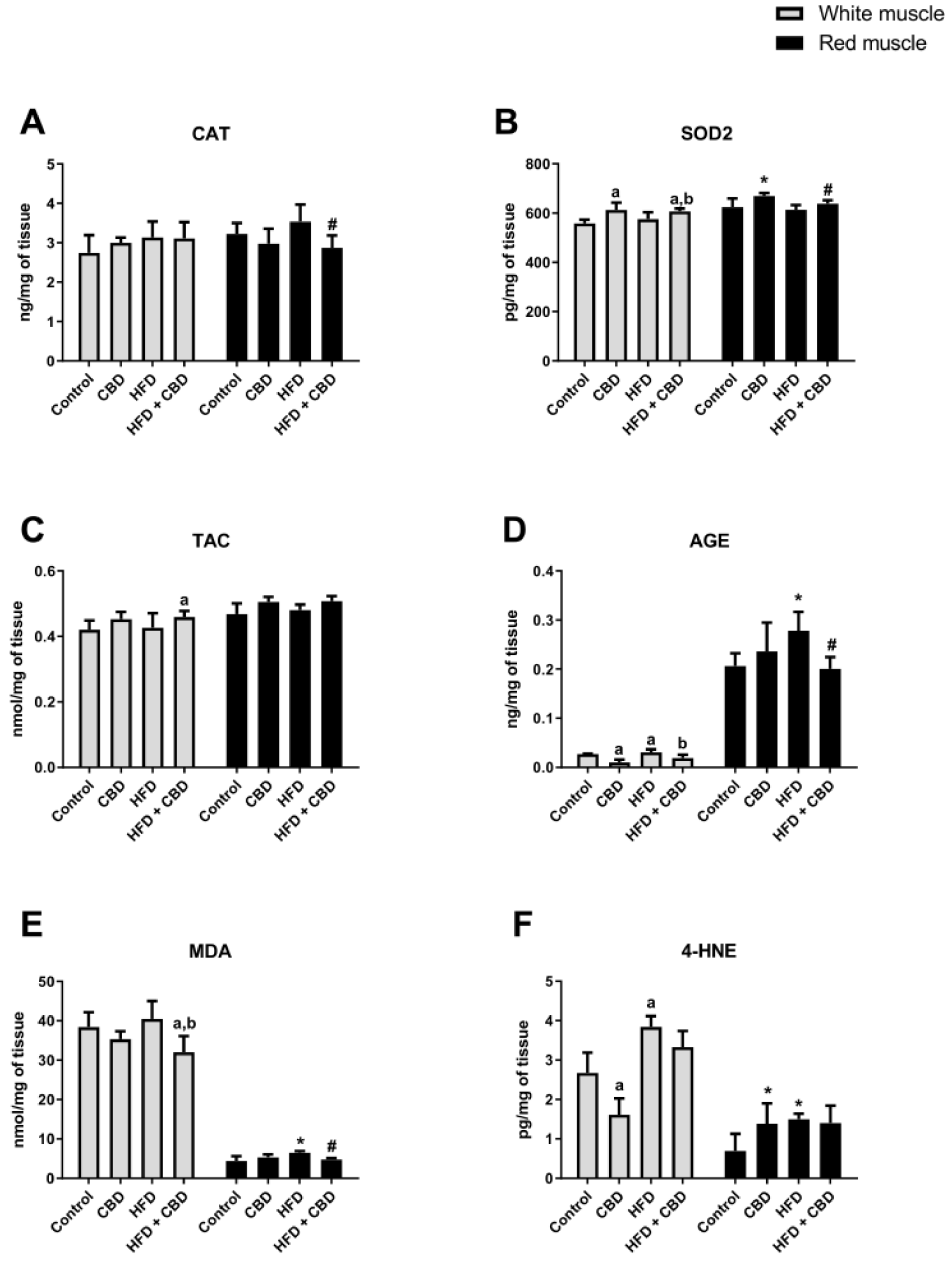
The effect of two-week cannabidiol (CBD) treatment on oxidative stress parameters, i.e., on (**A**) catalase (CAT), (**B**) superoxide dismutase 2 (SOD2), (**C**) total antioxidant capacity (TAC), (**D**) advanced glycation end products (AGE), (**E**) malonyldialdehyde (MDA) and (**F**) 4-hydroxynonenal (4-HNE), in the white and red gastrocnemius muscles in the control (standard diet) and high-fat diet (HFD) groups. The data are expressed as mean values ± SD, *n* = 6 in each group. ^a^ *p* < 0.05 indicates a significant difference: the control group vs. the examined group in the white gastrocnemius muscle; ^b^ *p* < 0.05 indicates a significant difference: HFD vs. HFD + CBD in the white gastrocnemius muscle. * *p* < 0.05 indicates a significant difference: the control group vs. the examined group in the red gastrocnemius muscle; ^#^ *p* < 0.05 indicates a significant difference: HFD vs. HFD + CBD in the red gastrocnemius muscle.

In the experimental model of HFD-induced obesity, we noticed significantly decreased CAT values after two-week CBD administration in the rats fed the high-fat diet (−12.8%, *p* < 0.05, vs. the HFD group; Figure 3A) only in the red gastrocnemius muscle. Concomitantly, we did not observe any changes in catalase concentrations in the white gastrocnemius muscle (*p* > 0.05; Figure 3A). Additionally, two-week CBD treatment of the rats fed a standard or high-fat diet resulted in a pronounced increase in SOD2 concentrations (+9.9% and +8.8%, respectively; *p* < 0.05; Figure 3B) in the white gastrocnemius muscle in comparison with the controls. Similar effects of CBD administration we reported in the red gastrocnemius muscle in the rats fed a standard chow (+7.4%, *p* < 0.05, vs. the control group; Figure 3B). Importantly, in both white and red skeletal muscles, chronic CBD administration caused a considerable increment in SOD2 levels in the rats subjected to the high-fat diet (+7.1% and +3.9%, *p* < 0.05; Figure 3B) compared to the HFD group alone. With respect to the total antioxidant capacity, we observed a substantial increase in the HFD-fed group in the chronic presence of CBD (+9.1%, *p* < 0.05, vs. the control group; Figure 3C) only in the white skeletal muscle. Moreover, in the same muscle, in the standard chow-fed group, after CBD treatment, we observed a trend towards an increase in the TAC (*p* = 0.0661; Figure 3C). Simultaneously, we did not notice any significant alterations in TAC levels in the red gastrocnemius muscle (*p* > 0.05; Figure 3C). Furthermore, as expected, the high-fat diet feeding induced a greater intramuscular AGE concentration in both white and red skeletal muscles (+13.3% and +32.1%, respectively; *p* < 0.05, vs. the control group; Figure 3D), which was subsequently decreased by chronic CBD administration (−38.8% and −28.0%, respectively; *p* < 0.05, vs. the HFD group; Figure 3D). A similar effect of CBD treatment was also observed in the rats fed a standard diet (−64.5%, *p* < 0.05; Figure 3D) in the white gastrocnemius muscle compared to the control group. Consequently, the MDA content was significantly increased in the animals fed an HFD in the red gastrocnemius muscle (+45.6%, *p* < 0.05, vs. the control group; Figure 3E), which was further reduced after CBD administration (−26.9% *p* < 0.05, vs. the HFD group; Figure 3E). Regarding the white skeletal muscle, the MDA concentration was considerably decreased in the presence of CBD during high-fat diet administration (−16.7%, *p* < 0.05, vs. the control group; −22.9%, *p* < 0.05, vs. the HFD group; Figure 3E). Similarly, high-fat diet feeding resulted in a substantial increase in the 4-HNE levels in both white and red gastrocnemius muscles (+43.7% and +115.7%, respectively; *p* < 0.05; Figure 3F) in comparison with the control conditions. In addition, we noticed a significantly decreased content of 4-HNE after prolonged CBD treatment in the rats fed a standard chow (−39.8%, *p* < 0.05, vs. the control group; Figure 3F) in the white gastrocnemius muscle, whereas, on the contrary, we observed a pronounced increase in the 4-HNE concentration in the same experimental group in the red skeletal muscle (+124.6%, *p* < 0.05; Figure 3F) compared to the control rats. 

### 3.3. Effect of Two-Week CBD Treatment on the Total Intramuscular Expression of Proteins Involved in the Inflammatory Pathway in the White and Red Skeletal Muscles of Rats Subjected to Standard and High-Fat Diets

**Figure 4:**
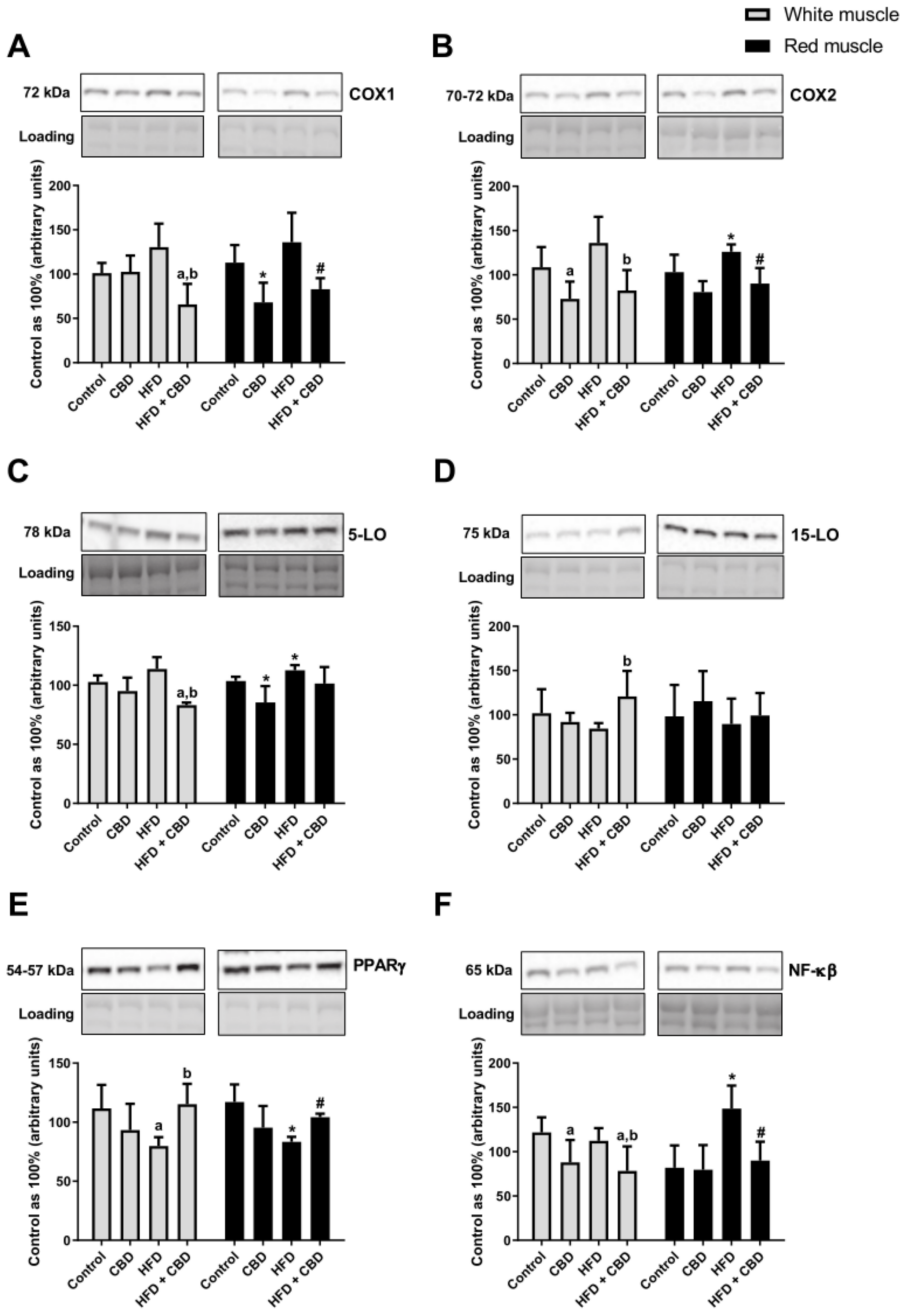
The total expression of proteins involved in the eicosanoid synthesis pathway, i.e., of (**A**) cyclooxygenase-1 (COX-1), (**B**) cyclooxygenase-2 (COX-2), (**C**) 5-lipoxygenase (5-LO), (**D**) 15-lipoxygenase (15-LO) and (**E**) peroxisome proliferator-activated receptor gamma (PPARγ), as well as total expression of (**F**) nuclear factor-κB (NF-κB) in the white and red gastrocnemius muscles in the control (standard diet) and high-fat diet (HFD) groups. The total expressions of the abovementioned proteins are presented as percentage differences compared to the control group which was set as 100%. The data are expressed as mean values ± SD, *n* = 6 in each group. ^a^ *p* < 0.05 indicates a significant difference: the control group vs. the examined group in the white gastrocnemius muscle; ^b^ *p* < 0.05 indicates a significant difference: HFD vs. HFD + CBD in the white gastrocnemius muscle. * *p* < 0.05 indicates a significant difference: the control group vs. the examined group in the red gastrocnemius muscle; ^#^ *p* < 0.05 indicates a significant difference: HFD vs. HFD + CBD in the red gastrocnemius muscle.

**Figure 5:**
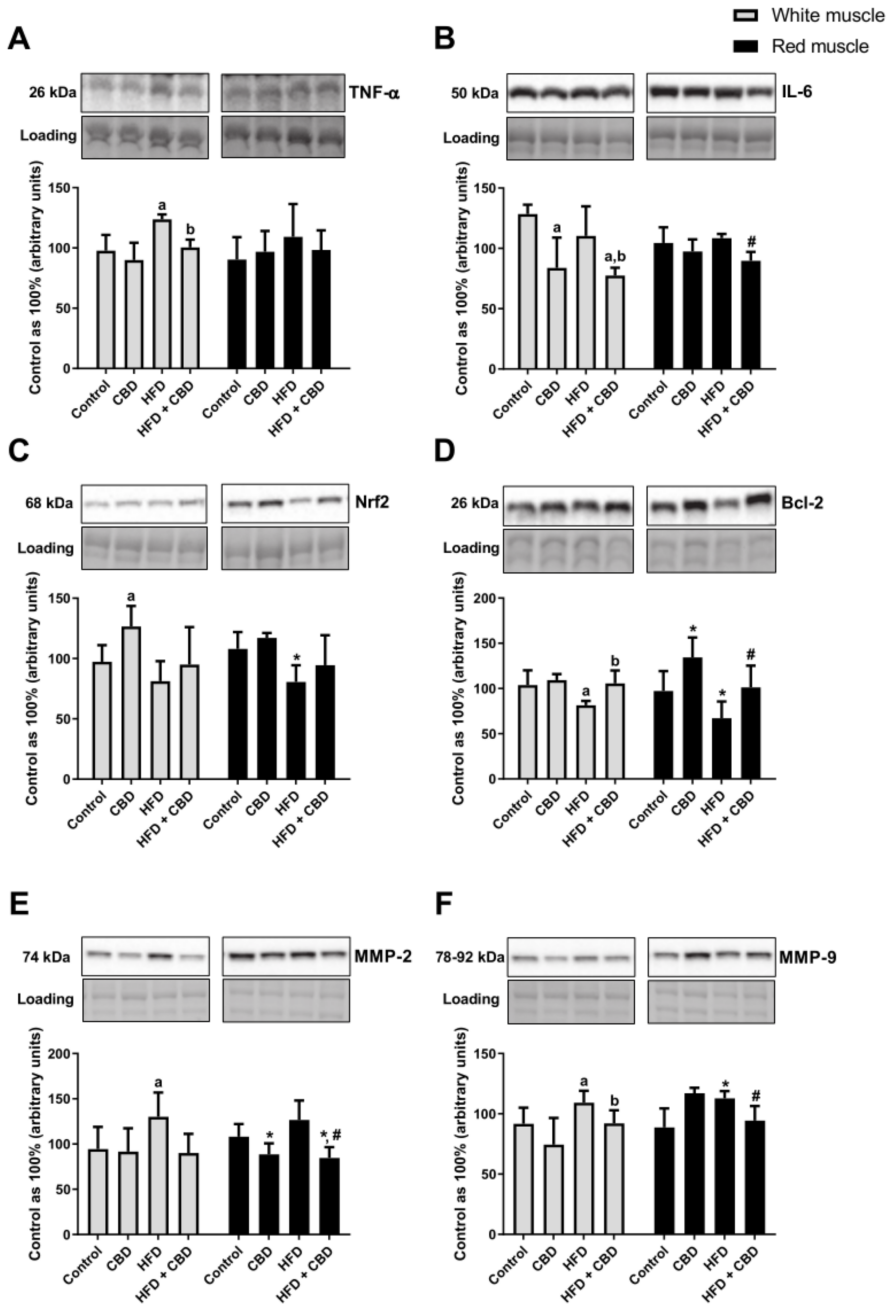
Intramuscular changes in the total expression of proteins involved in the inflammatory pathway, i.e., of (**A**) tumor necrosis factor α (TNF-α), (**B**) interleukin 6 (IL-6), as well as (**C**) nuclear factor erythroid 2-related factor 2 (Nrf2), (**D**) B cell lymphoma 2 (Bcl-2), (**E**) matrix metalloproteinase-2 (MMP-2) and (**F**) matrix metalloproteinase-9 (MMP-9) in the white and red gastrocnemius muscles in the control (standard diet) and high-fat diet (HFD) groups. The total expressions of the abovementioned proteins are presented as percentage differences compared to the control group which was set as 100%. The data are expressed as mean values ± SD, *n* = 6 in each group. ^a^ *p* < 0.05 indicates a significant difference: the control group vs. the examined group in the white gastrocnemius muscle; ^b^ *p* < 0.05 indicates a significant difference: HFD vs. HFD + CBD in the white gastrocnemius muscle. ^*^ *p* < 0.05 indicates a significant difference: the control group vs. the examined group in the red gastrocnemius muscle; ^#^ *p* < 0.05 indicates a significant difference: HFD vs. HFD + CBD in the red gastrocnemius muscle.

Compared to the control group, the rats fed a high-fat diet were characterized by a substantial increase in the total expression of COX2 and 5-LO in the red gastrocnemius muscle (+22.3% and +8.9%, respectively; *p* < 0.05; Figure 4B,C). Moreover, during HFD administration, we observed a trend towards an increase in the COX1 expression (*p* = 0.0590, vs. the control group; Figure 4A) in the white gastrocnemius muscle. Most importantly, two-week CBD injections in the high-fat diet group resulted in a considerable reduction of the total intramuscular expression of the proteins involved in the inflammatory pathway in both white and red gastrocnemius muscles, i.e., of COX1 (−49.5% and −39.0%, respectively; *p* < 0.05; Figure 4A) and COX2 (−39.5% and −28.4%, respectively; *p* < 0.05; Figure 4B) compared to the HFD alone. Similar effects of CBD treatment in the HFD-fed rats we reported in the case of the total intramuscular 5-LO expression in the white gastrocnemius muscle (−19.1%, *p* < 0.05, vs. the control group; −26.9%, *p* < 0.05, vs. the HFD group; Figure 4C). Simultaneously, we noticed a markedly declined total expression of COX1 and 5-LO in the rats fed a standard chow with the chronic presence of CBD in the red skeletal muscle (−32.8% and −17.5%, *p* < 0.05, vs. the control group; Figure 4A,C). Concomitantly, in the white skeletal muscle, we observed that CBD treatment of the rats subjected to an HFD substantially increased the total expression of anti-inflammatory 15-LO (+43.2%, *p* < 0.05; Figure 4D) in comparison with the HFD group alone. As presented in Figure 4, intramuscular PPARγ expression decreased considerably during the course of a high-fat diet in the white (−28.5%, *p* < 0.05; Figure 4E) and red (−28.8%, *p* < 0.05; Figure 4E) skeletal muscles in comparison with the control rats. However, prolonged CBD administration in the high-fat diet rats resulted in pronounced restoration of the total expression of PPARγ in both muscle types (+44.2% and +25.0%, respectively; *p* < 0.05; Figure 4E) compared to the HFD group. Furthermore, the group receiving a high-fat diet demonstrated a significant elevation in the total muscular expression of NF-κB in the red gastrocnemius muscle (+81.5%, *p* < 0.05, vs. the control group; Figure 4F). We also found that in the same muscle type, chronic CBD treatment in the HFD-fed rats substantially reduced the expression of NF-κB (−39.4%, *p* < 0.05; Figure 4F) and IL-6 (−17.2%, *p* < 0.05; Figure 5B) in comparison with the HFD group. Similarly, in the white gastrocnemius muscle of the rats subjected to the standard or high-fat chow administration, we observed a significant decrease in the total expression of NF-κB after CBD injections (−27.8% and −35.8%, respectively, *p* < 0.05, vs. the control group; −30.3%, *p* < 0.05, vs. the HFD group; Figure 4F) and IL-6 (−34.7% and −39.7%, respectively, *p* < 0.05, vs. the control group; −29.9%, *p* < 0.05, vs. the HFD group; Figure 5B). In the case of TNF-α, we reported that the rats subjected to an HFD presented a substantially increased expression (+26.6%, *p* < 0.05, vs. the control group; Figure 5A), which was further decreased by CBD treatment (−18.7%, *p* < 0.05, vs. the HFD group; Figure 5A) in the white gastrocnemius muscle. Concomitantly, we did not notice any significant changes in TNF-α expression in the oxidative muscle (*p* > 0.05; Figure 5A). Moreover, we observed that high-fat diet feeding reduced the total expression of Nrf2 in the red skeletal muscle (−25.3%, *p* < 0.05; Figure 5C) and Bcl-2 in both muscle types (−21.5% and −31.3%, respectively; *p* < 0.05; Figure 5D) compared to the controls. Interestingly, Nrf2 expression considerably increased in the standard chow-fed group after two-week CBD injections (+30.1%, *p* < 0.05, vs. the control group; Figure 5C) in the white gastrocnemius muscle. Similar effects of CBD treatment compared to the control conditions were observed with respect to the total expression of Bcl-2 (+38.1%, *p* < 0.05; Figure 5D) in the red skeletal muscle. Concomitantly, we also noticed a substantial increase in the total Bcl-2 expression in the white and red gastrocnemius muscles of the HFD group after CBD administration (+29.5% and +50.9%, respectively; *p* < 0.05; Figure 5D) in comparison with the HFD group alone. Moreover, in both muscle types, we reported that high-fat diet feeding significantly increased the total expression of MMP-2 (+37.9%, *p* < 0.05; Figure 5E) and MMP-9 (+19.1% and +27.2%, respectively; *p* < 0.05; Figure 5F) compared to the respective controls. Simultaneously, in the red gastrocnemius muscle of the rats subjected to the standard or high-fat chow administration, we observed a significant decrease in the total expression of MMP-2 after CBD administration (−21.6% and −21.8%, respectively, *p* < 0.05, vs. the control group; −33.2%, *p* < 0.05, vs. the HFD group; Figure 5E). Most importantly, CBD injections in the HFD-fed rats considerably decreased the expression of MMP-9 in both white (−21.5%, *p* < 0.05; Figure 5F) and red (−16.4%, *p* < 0.05; Figure 5F) gastrocnemius muscles in comparison with the HFD group. 

## 4. Discussion

Our research has shown that an obesogenic high-fat diet influences the fatty acid composition in the white and red skeletal muscles as well as contributes to the development of oxidative stress and local inflammation. Moreover, for the first time, we evaluated the impact of CBD on the n-6/n-3 PUFA ratio in different lipid fractions together with oxidative stress parameters and inflammatory pathways in a rat model of HFD-induced obesity. It is worth emphasizing, that the present study assessed the effect of CBD on the abovementioned levels in relation to the metabolism and oxidative capacity of the two types of skeletal muscle tissues (namely, with dominant oxidative vs. glycolytic metabolism).

Our data showed that HFD feeding caused an imbalance in the n-6/n-3 PUFA ratio, which was further effectively improved by two-week CBD administration. Specifically, a shift in the n-6/n-3 PUFA equilibrium in favor of anti-inflammatory n-3 PUFAs, especially in the red gastrocnemius muscle, which is probably due to the fact that the fibers of this type use FAs as an energy substrate for oxidative metabolism. In recent years, several studies have reported that n-6 and n-3 PUFAs have an opposite effect on IR and body homeostasis [33,34]. It is postulated that n-3 PUFAs, including ALA (C18:3; a precursor of EPA), EPA (C20:5) and DHA (C22:6), attenuate the development of IR via reducing inflammation, while n-6 PUFAs, primarily AA (C20:4) and its precursor LA (C18:2), promote emergence of IR [35]. The latest evidence also indicates that especially AA plays a key role in the inflammatory process and is associated with various metabolic diseases [11]. Therefore, it seems reasonable to control the dietary ratios of n-6/n-3 PUFAs in order to ameliorate obesity-related IR, which is beneficial for protection against chronic and metabolic diseases. In our study, as expected, we demonstrated that feeding rats an HFD resulted in an increased accumulation of fatty acids in the red gastrocnemius muscle in various lipid fractions (i.e., FFA, DAG, TAG and PL) with a concomitant shift in the n-6/n-3 PUFA balance towards n-6 PUFAs, mostly due to the elevated content of LA and AA. On the other hand, in the case of the white gastrocnemius muscle, we observed similar effects only in the TAG fraction, whereas in the FFA, DAG and PL lipid pools, there was a slight reduction in the n-6/n-3 PUFA ratio. Increased fatty acids accumulation in the TAG fraction results from the fact that this lipid pool serves as an energy substrate store [36]. In the red gastrocnemius muscle, the increase of the n-6/n-3 levels in response to HFD feeding turned out to be more pronounced than in the white type, which is consistent with the metabolism and use of FAs as an energy substrates by oxidative fibers [37]. Simultaneously, lipid oversupply induced an elevation in the proportion of circulating n-6 PUFAs to n-3 PUFAs in both plasma FFA and TAG, which was in line with the findings of other researchers [38]. Interestingly, in our experiment, we found that the observed increase in the ratio of n-6 to n-3 PUFAs in the plasma FFA fraction resulted from a significant increase in the LA and AA contents, whereas with regard to plasma TAG, this shift was due to marked reduction in the amount of n-3 PUFAs, i.e., of ALA, EPA and DHA. However, the major finding of the present study is that two-week CBD treatment could effectively lower the n-6/n-3 PUFA ratio by shifting the equilibrium in favor of n-3 fatty acids and increasing their incorporation into different lipid fractions. In the red gastrocnemius muscle, we noticed significantly elevated n-3 PUFAs, mainly EPA and DHA, along with a substantial reduction in the LA content (n-6 PUFA) in all the examined lipid fractions of the animals chronically treated with CBD during an HFD course. It is worth noting that tissue LA is considered an obesity-promoting FA since it serves as a precursor to AA, which is a substrate in the synthesis of AEA and 2-AG [39]. Thus, the increased synthesis of these ECs largely explains the ability of LA to produce obesogenic effects. Moreover, in our study, we revealed that the AA concentration was significantly enhanced in the FFA, DAG and PL lipid pools in the rats fed both the standard chow and the HFD after CBD injections in the muscle with oxidative metabolism, which may have resulted from the overactivation of the eCBome in obesity and its additional stimulation by CBD [40]. Surprisingly, in the white skeletal muscle, CBD caused a pronounced elevation in the n-6/n-3 PUFA ratio in the FFA and DAG fractions of the rats fed an HFD resulting from increased incorporation of LA and AA. Conversely, in the TAG and PL lipid pools of the same muscle type, we observed an improvement in the n-6/n-3 PUFA imbalance after CBD administration, which was associated with a greater increase in the content of essential n-3 PUFA species, mainly, the amount of DHA. These findings demonstrate that CBD may play a pivotal role in the control of the membrane fatty acid content through the reduction of substrates for ECs and inflammatory molecules synthesis as well as improvement in the tissue n-3 essential fatty acids concentration. Additionally, an increasing amount of evidence suggests that n-3 PUFAs improve insulin sensitivity in skeletal muscle and liver in different animal models, e.g., in diet-induced obese mice and rats [41,42,43,44]. 

Moreover, the following study showed a link between the shifts in the n-6/n-3 PUFA balance towards n-6 PUFAs and the increasing inflammatory response in fatty acid oversupply conditions. N-6 PUFAs, particularly AA, are considered to be the most important source of precursors for the synthesis of ECs and proinflammatory eicosanoids, including prostaglandins, leukotrienes, thromboxanes and hydroxyeicosatetraenoic acids (HETEs), which intensify the inflammatory signaling cascade [45]. Consistently, in our research, we observed a substantial elevation in the total expression of enzymes catalyzing the production of the abovementioned lipid mediators, such as cyclooxygenase 1 (COX1), cyclooxygenase 2 (COX2) and 5-lipoxygenase (5-LO) in both white and red gastrocnemius muscles of the rats subjected to an HFD for seven weeks. Simultaneously, two-week CBD administration significantly reduced their skeletal muscle expression in the same HFD group, which is in line with previous findings describing the inhibitory action of CBD on COX1, COX2 and 5-LO in an in vitro model (human colon adenocarcinoma cell line HT29) [46,47]. Additionally, in the white skeletal muscle, CBD treatment caused a substantial elevation of anti-inflammatory 15-lipoxygenase (15-LO) in the HFD-fed rats [48]. Furthermore, in our study, we noticed a considerably elevated PPARγ expression after CBD administration in the rats fed an HFD regardless of the metabolism of a given muscle (oxidative vs. glycolytic). We can assume that the decreased expression of proinflammatory enzymes in muscles after CBD injections may be indirectly related to an increase in the expression of PPARγ, whose CBD is an agonist [49]. Moreover, some studies have shown that n-3 PUFAs can increase PPARγ expression in vitro (e.g., the HaCaT keratinocyte cell line [50]) and in vivo (e.g., C57BL/6 mice [51]) as natural ligands of this transcription factor; therefore, we can hypothesize that anti-inflammatory properties of n-3 PUFAs may be related to the PPARγ upregulation. Accordingly, Hou et al. demonstrated that PPARγ activation results in proteasomal degradation of the p65 subunit of nuclear factor-κB (NF-κB) which inhibits expression of the genes involved in inflammation (e.g., COX2) as well as of some proinflammatory mediators (e.g., TNF-α and IL-6) [52]. The results obtained in our experiment are in agreement with this statement; we noticed a pronounced reduction in the total muscular expression of NF-κB after two-week CBD administration in both white and red gastrocnemius muscles of high-fat diet-fed rats in comparison with the HFD group alone. Concomitantly, similar alterations caused by CBD in the expression of inflammatory mediators, such as IL-6, were noticeable in the muscles with predominant oxidative and glycolytic metabolism of the rats subjected to a high-fat diet. Moreover, PPARγ also collaborates with another transcription factor, nuclear factor erythroid 2-related factor 2 (Nrf2), which regulates many cytoprotective genes such as antioxidant proteins, including CAT and SOD2 (Scheme 1) [53,54]. In the studies conducted by Huang et al., the researchers revealed that PPARγ was activated by transcriptional activity of Nrf2, which was also confirmed by several other studies [55,56]. However, some lines of evidence indicated the possibility of direct activation of the Nrf2 pathway by PPARγ, which suggests that the Nrf2 and PPARγ pathways are related in a positive feedback loop [57]. Our research revealed that high-fat diet feeding in rats decreased the expression of Nrf2, which was further restored by two-week CBD administration. Intriguingly, CBD also elevated the total Nrf2 expression in the rats fed a standard chow; however, changes reached the significant level only in the white gastrocnemius muscle. In in vitro studies, Mammana et al. also displayed that CBD in a dose of 2.5 and 5 μM substantially upregulated the expression of Nrf2 in NSC-34 motor neurons with lipopolysaccharide (LPS)-induced inflammation [58]. In the same experiment, the researchers pointed out a considerable elevation in expression of B cell lymphoma 2 (Bcl-2), an anti-apoptotic protein, following CBD treatment (5 μM), which is consistent with the results obtained in our experiment, where we observed a significant increase in the Bcl-2 protein in both muscle types in the rats fed an HFD [58]. Additionally, in our study, we reported that the rats subjected to high-fat diet feeding exhibited a substantially increased muscular expression of matrix metalloproteinases (MMPs) MMP-2 and MMP-9 which belong to the enzymes that promote the breakdown and remodeling of tissues as well as the inflammatory process and related migration of white blood cells [59]. It is worth emphasizing that two-week CBD administration considerably decreased the total MMP-2 and MMP-9 expression in the same experimental animals in both white and red gastrocnemius muscles. Similar results were obtained by Elbaz et al. in a study where CBD treatment (6 μM) reduced the activities of MMP-2 and MMP-9 in triple-negative breast cancer (TNBC) cell lines [60]. This finding further implies CBD’s potentially beneficial cellular protective effects.

In obesity, the excessive amount of fat exceeds the ability of skeletal muscles to oxidize this energy substrate, which leads to the development of lipotoxicity and disruption of the physiological muscle function, including impaired insulin signaling. These harmful effects of accumulated lipid intermediates were partially attributed to the ROS/RNS overproduction that, together with attenuated antioxidant defense capacity, elicits systemic oxidative stress [61]. It is worth noting that skeletal muscles, due to different fiber type composition (oxidative vs. glycolytic), vary in susceptibility to oxidative stress-induced damage since they have different contents of mitochondria that generate ROS and distinct antioxidative capacity [62]. Myocytes have their own antioxidant system containing various antioxidant enzymes including CAT, SOD2 and GPx [62]. In the present study, we reported that feeding rats an HFD did not produce any alterations in the muscular CAT, SOD2 and total antioxidant capacity (TAC) levels in the skeletal muscles irrespective of presented metabolism. However, chronic CBD exposure of the rats subjected to an HFD reduced the CAT level only in the red gastrocnemius muscle and enhanced the TAC in the white skeletal muscle. On the other hand, CBD administration notably increased the level of SOD2 in skeletal muscles with a different fiber type composition in the rats fed either a standard or an HFD. This was likely due to the fact that CBD was found to increase the mRNA level of SOD2 [24]; hence, we can assume that one of the mechanisms responsible for this CBD’s action is Nrf2 signaling pathway activation. Additionally, it should be remarked that CBD has the ability to reduce the oxidative modifications of lipids that result from oxidation of membrane PUFAs by ROS [63]. This reaction generates unsaturated aldehydes, including malonyldialdehyde (MDA) and 4-hydroxynonenal (4-HNE), which are highly reactive and can lead to cellular dysfunctions. Our results, as expected, confirmed that rats in the state of HFD-induced obesity exhibited considerably elevated concentrations of both MDA and 4-HNE in the white and red gastrocnemius muscles. As regards the involvement of the muscle type in the effect of HFD feeding, the white gastrocnemius muscle demonstrated much higher concentrations of the abovementioned lipid peroxidation byproducts. This is probably caused by the structure of the white skeletal muscle which consists mainly of glycolytic fibers, using FA as an energy substrate to a small extent [61]. Most importantly, in the conducted experiment, we reported that treatment of the HFD-fed rats with CBD prevents the accumulation of MDA in both fast- and slow-twitch skeletal muscles. Furthermore, the 4-HNE concentration was decreased after prolonged CBD administration in the rats fed a standard chow or HFD in the white gastrocnemius muscle; however, these changes reached statistical significance only in the group of rats fed a standard diet. In addition, in the following study, we also evaluated the impact of CBD on advanced glycation end products (AGE) in conditions of chronic exposure to a high-fat diet. Not surprisingly, we reported that AGEs in rats on an HFD increased substantially in both muscle types, wherein the red gastrocnemius muscle exhibited intensified accumulation of AGEs in comparison with the white gastrocnemius muscle, which is consistent with results of other researchers [64]. This may be due to basal metabolism and the muscle’s adaptive response to overabundance of lipids during chronic exposure to an HFD. Noteworthily, two-week CBD administration to the high-fat diet fed animals caused a considerable reduction in the AGE concentration in both oxidative and glycolytic muscles. The mechanism of this effect is still not entirely clear but seems to involve multiple molecular pathways. Based on these findings, CBD could be a therapeutically useful agent in combating the effects of oxidative stress and inflammation associated with obesity and its metabolic disturbances.

## 5. Conclusions

Taken altogether, our results clearly demonstrated that an HFD promotes excessive accumulation of fatty acids in the skeletal muscle with a concomitant shift in the n-6/n-3 PUFA balance towards n-6 PUFAs, which is related to an increasing inflammatory response and oxidative stress and may contribute to obesity-associated onset of insulin resistance. Moreover, our data suggest that the impact of oxidative stress affects skeletal muscle function and metabolism when lipid FA overabundance is fiber type-specific. The major finding of the present study is that two-week CBD treatment effectively reduced the accumulation of fatty acids in muscular lipid pools and shifted the equilibrium of n-6/n-3 PUFAs in favor of anti-inflammatory n-3 PUFAs regardless of muscle metabolism. Moreover, CBD prevented generation of lipid peroxidation products, particularly in the glycolytic fibers. Concomitantly, it increased the antioxidative capacity of the muscles. Collectively, our observations emphasize the notion that chronic CBD administration may have a great therapeutic potential in the treatment of obesity-associated complications by alleviating inflammation and related lipid mediators as well as oxidative stress, which coincide with insulin resistance in obesity.

## Figures and Tables

**Table 1:** Polyunsaturated fatty acids (PUFA) content in the FFA fraction (nmol/g of wet tissue) in the white gastrocnemius muscle in the control (standard diet) and high-fat diet (HFD) groups after two-week cannabidiol (CBD) treatment.

Fatty Acid	Control	CBD	HFD	HFD + CBD
(18:2n6c)	57.59 ± 8.04	82.1 ± 14.10 ^a^	49.51 ± 10.53 ^a^	93.61 ± 7.54 ^a,b^
(20:4n6)	17.47 ± 1.77	26.69 ± 6.90 ^a^	15.70 ± 1.50 ^a^	47.80 ± 5.31 ^a,b^
(18:3n3)	6.63 ± 0.83	9.19 ± 2.02 ^a^	5.57 ± 0.89 ^a^	7.28 ± 0.84 ^b^
(20:5n3)	2.15 ± 0.36	1,89 ± 0.40	1.85 ± 0.43 ^a^	1.53 ± 0.28 ^a^
(22:6n3)	4.81 ± 0.93	7.36 ± 1.73 ^a^	6.40 ± 1.01 ^a^	12.57 ± 1.29 ^a,b^
n-6 PUFAs	75.06 ± 9.31	106.76 ± 15.28 ^a^	65.21 ± 10.78 ^a^	142.87 ± 13.07 ^a,b^
n-3 PUFAs	13.67 ± 1.68	18.43 ± 3.83 ^a^	13.57 ± 1.63	21.32 ± 1.81 ^a,b^

The data are expressed as mean values ± SD, *n* = 10 in each group. ^a^ *p* < 0.05 indicates a significant difference: the control group vs. the examined group in the white gastrocnemius muscle; ^b^ *p* < 0.05 indicates a significant difference: HFD vs. HFD + CBD in the white gastrocnemius muscle; 18:2n6c (linoleic acid, LA); 20:4n6 (arachidonic acid, AA); 18:3n3 (α-linolenic acid, ALA); 20:5n3 (eicosapentaenoic acid, EPA); 22:6n3 (docosahexaenoic acid, DHA).

**Table 2:** Polyunsaturated fatty acids (PUFA) content in the FFA fraction (nmol/g of wet tissue) in the red gastrocnemius muscle in the control (standard diet) and high-fat diet (HFD) groups after two-week cannabidiol (CBD) treatment.

Fatty Acid	Control	CBD	HFD	HFD + CBD
(18:2n6c)	356.30 ± 78.40	495.83 ± 44.51 *	626.91 ± 53.59 *	582.61 ± 59.54 *
(20:4n6)	77.08 ± 9.82	109.19 ± 5.69 *	109.23 ± 5.12 *	153.63 ± 7.87 *^,#^
(18:3n3)	26.26 ± 5.10	35.10 ± 3.11 *	33.54 ± 4.00 *	31.08 ± 3.65 *
(20:5n3)	3.56 ± 0.82	4.71 ± 0.52 *	3.59 ± 0.62	4.12 ± 0.66 *^,#^
(22:6n3)	35.16 ± 8.30	46.17 ± 7.06 *	61.57 ± 8.15 *	71.30 ± 8.90 *^,#^
n-6 PUFAs	433.38 ± 87.32	606.71 ± 48.96 *	736.14 ± 57.79 *	739.06 ± 64.82 *
n-3 PUFAs	64.85 ± 13.92	85.98 ± 8.19 *	99.32 ± 11.71 *	109.76 ± 6.85 *

The data are expressed as mean values ± SD, *n* = 10 in each group. * *p* < 0.05 indicates a significant difference: the control group vs. the examined group in the red gastrocnemius muscle; ^#^ *p* < 0.05 indicates a significant difference: HFD vs. HFD + CBD in the red gastrocnemius muscle; 18:2n6c (linoleic acid, LA); 20:4n6 (arachidonic acid, AA); 18:3n3 (α-linolenic acid, ALA); 20:5n3 (eicosapentaenoic acid, EPA); 22:6n3 (docosahexaenoic acid, DHA).

**Table 3:** Polyunsaturated fatty acids (PUFA) content in the DAG fraction (nmol/g of wet tissue) in the white gastrocnemius muscle in the control (standard diet) and high-fat diet (HFD) groups after two-week cannabidiol (CBD) treatment.

Fatty Acid	Control	CBD	HFD	HFD + CBD
(18:2n6c)	41.10 ± 6.95	35.10 ± 3.93 ^a^	31.99 ± 7.50 ^a^	40.55 ± 7.49 ^b^
(20:4n6)	20.73 ± 2.65	27.46 ± 4.72 ^a^	16.61 ± 1.94 ^a^	47.76 ± 6.92 ^a,b^
(18:3n3)	4.06 ± 0.62	3.73 ± 0.48	3.36 ± 0.86 ^a^	3.07 ± 0.31 ^a^
(20:5n3)	1.37 ± 0.38	1.06 ± 0.30	1.29 ± 0.31	1.05 ± 0.19 ^a^
(22:6n3)	3.68 ± 0.53	3.77 ± 0.71	5.23 ± 0.97 ^a^	7.66 ± 0.92 ^a,b^
n-6 PUFAs	61.83 ± 9.13	62.56 ± 7.47	48.59 ± 7.61 ^a^	90.40 ± 13.61 ^a,b^
n-3 PUFAs	9.12 ± 1.23	8.56 ± 1.07	10.05 ± 2.22	12.07 ± 1.58 ^a,b^

The data are expressed as mean values ± SD, *n* = 10 in each group. ^a^ *p* < 0.05 indicates a significant difference: the control group vs. the examined group in the white gastrocnemius muscle; ^b^ *p* < 0.05 indicates a significant difference: HFD vs. HFD + CBD in the white gastrocnemius muscle; 18:2n6c (linoleic acid, LA); 20:4n6 (arachidonic acid, AA); 18:3n3 (α-linolenic acid, ALA); 20:5n3 (eicosapentaenoic acid, EPA); 22:6n3 (docosahexaenoic acid, DHA).

**Table 4:** Polyunsaturated fatty acids (PUFA) content in the DAG fraction (nmol/g of wet tissue) in the red gastrocnemius muscle in the control (standard diet) and high-fat diet (HFD) groups after two-week cannabidiol (CBD) treatment.

Fatty Acid	Control	CBD	HFD	HFD+CBD
(18:2n6c)	79.55 ± 22.95	74.12 ± 5.14	145.53 ± 26.29 *	135.21 ± 18.06 *
(20:4n6)	34.20 ± 5.68	44.42 ± 4.89 *	40.54 ± 2.42 *	61.16 ± 5.46 *^,#^
(18:3n3)	5.83 ± 1.61	5.82 ± 0.43	7.66 ± 0.51 *	8.45 ± 1.03 *^,#^
(20:5n3)	0.97 ± 0.18	1.36 ± 0.27 *	1.20 ± 0.61	1.75 ± 0.50 *^,#^
(22:6n3)	9.09 ± 2.84	9.91 ± 2.02	17.60 ± 2.67 *	21.02 ± 2.85 *^,#^
n-6 PUFAs	118.71 ± 32.16	118.54 ± 9.37	186.07 ± 26.82 *	202.15 ± 20.80 *
n-3 PUFAs	15.91 ± 4.80	16.81 ± 2.33	26.59 ± 2.56 *	31.22 ± 3.82 *^,#^

The data are expressed as mean values ± SD, *n* = 10 in each group. * *p* < 0.05 indicates a significant difference: the control group vs. the examined group in the red gastrocnemius muscle; ^#^ *p* < 0.05 indicates a significant difference: HFD vs. HFD + CBD in the red gastrocnemius muscle; 18:2n6c (linoleic acid, LA); 20:4n6 (arachidonic acid, AA); 18:3n3 (α-linolenic acid, ALA); 20:5n3 (eicosapentaenoic acid, EPA); 22:6n3 (docosahexaenoic acid, DHA).

**Table 5:** Polyunsaturated fatty acids (PUFA) content in the TAG fraction (nmol/g of wet tissue) in white gastrocnemius muscle in the control (standard diet) and high-fat diet (HFD) groups after two-week cannabidiol (CBD) treatment.

Fatty Acid	Control	CBD	HFD	HFD + CBD
(18:2n6c)	306.57 ± 97.30	295.80 ± 47.57	1340.05 ± 383.66 ^a^	879.74 ± 181.45 ^a,b^
(20:4n6)	17.51 ± 3.63	16.74 ± 2.51	28.85 ± 6.46 ^a^	30.21 ± 5.40 ^a^
(18:3n3)	31.39 ± 8.98	30.24 ± 5.17	74.72 ± 21.80 ^a^	61.33 ± 10.99 ^a^
(20:5n3)	4.63 ± 1.58	3.73 ± 1.12	3.95 ± 1.20	2.89 ± 1.48 ^a^
(22:6n3)	6.12 ± 2.12	9.18 ± 3.66 ^a^	11.56 ± 3.72 ^a^	16.64 ± 3.28 ^a,b^
n-6 PUFAs	324.08 ± 99.30	313.26 ± 49.97	1290.09 ± 434.82 ^a^	909.95 ± 183.75 ^a,b^
n-3 PUFAs	42.86 ± 11.48	42.64 ± 8.12	90.98 ± 28.53 ^a^	80.19 ± 11.43 ^a^

The data are expressed as mean values ± SD, *n* = 10 in each group. ^a^ *p* < 0.05 indicates a significant difference: the control group vs. the examined group in the white gastrocnemius muscle; ^b^ *p* < 0.05 indicates a significant difference: HFD vs. HFD + CBD in the white gastrocnemius muscle; 18:2n6c (linoleic acid, LA); 20:4n6 (arachidonic acid, AA); 18:3n3 (α-linolenic acid, ALA); 20:5n3 (eicosapentaenoic acid, EPA); 22:6n3 (docosahexaenoic acid, DHA).

**Table 6:** Polyunsaturated fatty acids (PUFA) content in the TAG fraction (nmol/g of wet tissue) in the red gastrocnemius muscle in the control (standard diet) and high-fat diet (HFD) groups after two-week cannabidiol (CBD) treatment.

Fatty Acid	Control	CBD	HFD	HFD + CBD
(18:2n6c)	775.65 ± 666.51	689.35 ± 226.72	1682.96 ± 820.25 *	1113.21 ± 353.52 ^#^
(20:4n6)	38.74 ± 26.02	36.78 ± 9.81	39.97 ± 17.23	39.70 ± 7.85
(18:3n3)	69.66 ± 55.34	66.62 ± 22.17	88.41 ± 37.28	79.79 ± 19.00
(20:5n3)	4.35 ± 1.60	4.15 ± 1.42	3.34 ± 1.02	4.91 ± 1.68 ^#^
(22:6n3)	12.72 ± 5.21	13.39 ± 3.49	33.26 ± 6.73 *	44.49 ± 4.76 *^,#^
n-6 PUFAs	965.12 ± 806.91	726.13 ± 236.31	1724.88 ± 837.21 *	1086.75 ± 311.93 ^#^
n-3 PUFAs	75.11 ± 49.38	83.67 ± 21.26	136.52 ± 53.96	129.19 ± 19.33

The data are expressed as mean values ± SD, *n* = 10 in each group. * *p* < 0.05 indicates a significant difference: the control group vs. the examined group in the red gastrocnemius muscle; ^#^ *p* < 0.05 indicates a significant difference: HFD vs. HFD + CBD in the red gastrocnemius muscle; 18:2n6c (linoleic acid, LA); 20:4n6 (arachidonic acid, AA); 18:3n3 (α-linolenic acid, ALA); 20:5n3 (eicosapentaenoic acid, EPA); 22:6n3 (docosahexaenoic acid, DHA).

**Table 7:** Polyunsaturated fatty acids (PUFA) content in the PL fraction (nmol/g of wet tissue) in the white gastrocnemius muscle in the control (standard diet) and high-fat diet (HFD) groups after two-week cannabidiol (CBD) treatment.

Fatty Acid	Control	CBD	HFD	HFD + CBD
(18:2n6c)	3804.57 ± 232.32	3562.35 ± 603.79	2302.71 ± 204.26^a^	2770.34 ± 572.05 ^a,b^
(20:4n6)	4249.78 ± 200.32	4074.72 ± 448.51	4168.76 ± 294.02	5146.26 ± 568.67 ^a,b^
(18:3n3)	45.76 ± 6.35	47.49 ± 3.80	25.67 ± 2.46 ^a^	28.83 ± 2.45 ^a,b^
(20:5n3)	49.26 ± 5.52	46.56 ± 5.41	26.23 ± 3.09 ^a^	30.88 ± 5.25 ^a,b^
(22:6n3)	1563.04 ± 119.25	1698.80 ± 44.15	1761.42 ± 265.15 ^a^	2644.32 ± 420.35 ^a,b^
n-6 PUFAs	8054.35 ± 342.47	7925.37 ± 456.35	6471.47 ± 344.45 ^a^	7622.72 ± 647.02 ^b^
n-3 PUFAs	1658.06 ± 122.19	1877.10 ± 694.05	1813.33 ± 266.27	2801.95 ± 712.31 ^a,b^

The data are expressed as mean values ± SD, *n* = 10 in each group. ^a^ *p* < 0.05 indicates a significant difference: the control group vs. the examined group in the white gastrocnemius muscle; ^b^ *p* < 0.05 indicates a significant difference: HFD vs. HFD + CBD in the white gastrocnemius muscle; 18:2n6c (linoleic acid, LA); 20:4n6 (arachidonic acid, AA); 18:3n3 (α-linolenic acid, ALA); 20:5n3 (eicosapentaenoic acid, EPA); 22:6n3 (docosahexaenoic acid, DHA).

**Table 8:** Polyunsaturated fatty acids (PUFA) content in the PL fraction (nmol/g of wet tissue) in the red gastrocnemius muscle in the control (standard diet) and high-fat diet (HFD) groups after two-week cannabidiol (CBD) treatment.

Fatty Acid	Control	CBD	HFD	HFD + CBD
(18:2n6c)	6016.99 ± 705.69	7281.82 ±495.03 *	6140.77 ± 331.08	7653.15 ± 440.06 *^,#^
(20:4n6)	3922.92 ± 371.42	4684.31 ±184.04 *	4765.12 ± 384.66 *	6019.30 ± 184.80 *^,#^
(18:3n3)	71.94 ± 7.37	79.89 ± 2.95 *	41.45 ± 4.01 *	48.43 ± 6.68 *^,#^
(20:5n3)	58.99 ± 5.93	54.48 ± 4.28	41.21 ± 3.97 *	43.74 ± 2.36 *
(22:6n3)	2283.89 ± 382.17	3793.13 ±419.76 *	3052.58 ± 291.44 *	4341.88 ± 260.71 *^,#^
n-6 PUFAs	9939.90 ± 907.86	11,884.97 ± 503.74 *	11,008.32 ± 512.03 *	13,672.45 ± 592.99 *^,#^
n-3 PUFAs	2419.60 ± 397.65	3857.01 ± 450.31 *	3140.37 ± 286.37 *	4471.28 ± 250.34 *^,#^

The data are expressed as mean values ± SD, *n* = 10 in each group. * *p* < 0.05 indicates a significant difference: the control group vs. the examined group in the red gastrocnemius muscle; ^#^ *p* < 0.05 indicates a significant difference: HFD vs. HFD + CBD in the red gastrocnemius muscle; 18:2n6c (linoleic acid, LA); 20:4n6 (arachidonic acid, AA); 18:3n3 (α-linolenic acid, ALA); 20:5n3 (eicosapentaenoic acid, EPA); 22:6n3 (docosahexaenoic acid, DHA).

**Table 9:** Polyunsaturated fatty acids (PUFA) content in the plasma FFA fraction (nmol/mL of plasma) in the control (standard diet) and high-fat diet (HFD) groups after two-week cannabidiol (CBD) treatment.

Fatty Acid	Control	CBD	HFD	HFD + CBD
18:2n6c	55.06 ± 12.31	83.88 ± 23.49 ^a^	92.76 ± 25.91 ^a^	147.53 ± 40.28 ^a,b^
20:4n6	8.16 ± 1.68	15.36 ± 2.17 ^a^	16.37 ± 4.24 ^a^	17.34 ± 4.88 ^a^
18:3n3	6.39 ± 1.75	9.18 ± 2.54 ^a^	7.48 ± 1.73	12.50 ± 3.72 ^a,b^
20:5n3	0.40 ± 0.08	0.49 ± 0.13 ^a^	0.23 ± 0.05 ^a^	0.29 ± 0.08 ^a,b^
22:6n3	2.14 ± 0.58	4.30 ± 1.16 ^a^	2.82 ± 0.67 ^a^	3.53 ± 1.28 ^a,b^
n-6 PUFAs	63.22 ± 13.49	100.93 ± 26.71 ^a^	109.13 ± 28.51 ^a^	157.30 ± 40.34 ^a,b^
n-3 PUFAs	8.93 ± 2.35	14.49 ± 3.78 ^a^	10.14 ± 2.58	16.32 ± 4.81 ^a,b^

The data are expressed as mean values ± SD, *n* = 10 in each group. ^a^ *p* < 0.05 indicates a significant difference: the control group vs. the examined group; ^b^ *p* < 0.05 indicates a significant difference: HFD vs. HFD + CBD; 18:2n6c (linoleic acid, LA); 20:4n6 (arachidonic acid, AA); 18:3n3 (α-linolenic acid, ALA); 20:5n3 (eicosapentaenoic acid, EPA); 22:6n3 (docosahexaenoic acid, DHA).

**Table 10:** Polyunsaturated fatty acids (PUFA) content in the plasma TAG fraction (nmol/mL of plasma) in the control (standard diet) and high-fat diet (HFD) groups after two-week cannabidiol (CBD) treatment.

Fatty Acid	Control	CBD	HFD	HFD + CBD
18:2n6c	1746.27 ± 258.44	1633.25 ± 445.53	1660.73 ± 964.61	1764.09 ± 408.46
20:4n6	126.75 ± 19.27	136.13 ± 19.36	132.11 ± 24.99	107.80 ± 18.37 ^b^
18:3n3	183.83 ± 40.07	176.14 ± 66.09	133.72 ± 95.65 ^a^	151.86 ± 53.02 ^b^
20:5n3	32.35 ± 6.81	32.34 ± 7.05	11.10 ± 2.69 ^a^	12.49 ± 3.11 ^a^
22:6n3	105.50 ± 21.13	129.68 ± 18.44 ^a^	46.35 ± 8.78 ^a^	55.49 ± 16.04 ^a^
n-6 PUFAs	1880.31 ± 264.08	1689.88 ± 377.78	2130.27 ± 992.20	1882.45 ± 426.13
n-3 PUFAs	327.95 ± 56.27	340.65 ± 71.39	151.91 ± 40.00 ^a^	221.56 ± 70.30 ^a,b^

The data are expressed as mean values ± SD, *n* = 10 in each group. ^a^ *p* < 0.05 indicates a significant difference: the control group vs. the examined group; ^b^ *p* < 0.05 indicates a significant difference: HFD vs. HFD + CBD; 18:2n6c (linoleic acid, LA); 20:4n6 (arachidonic acid, AA); 18:3n3 (α-linolenic acid, ALA); 20:5n3 (eicosapentaenoic acid, EPA); 22:6n3 (docosahexaenoic acid, DHA).

## Data Availability

The data presented in this study are available on request from the corresponding author.

## Supplementary Materials

The following are available online at https://www.mdpi.com/article/10.3390/nu13051603/s1, Supplementary File S1: The images of whole gels showing the total protein loading and the expression of selected proteins in the white and red gastrocnemius muscles in the control (standard diet) and high-fat diet (HFD) groups.
