# Supplementary material for: Attenuation of Oxidative Stress and Inflammatory Response by Chronic Cannabidiol Administration Is Associated with Improved n-6/n-3 PUFA Ratio in the White and Red Skeletal Muscle in a Rat Model of High-Fat Diet-Induced Obesity
Source: Nutrients. 2021 May 11;13(5):1603. doi: 10.3390/nu13051603 (PMC8151284; doi:10.3390/nu13051603)
Supplement: Supplementary file 1 [file nutrients-13-01603-s001.zip › nutrients-1177769-SI.pdf]

Loading order in all Western blots: Control, HFD, CBD, HFD+CBD.

On the left are stain-free gels showing the total protein loading used for normalization.

On the right are corresponding membranes with the detected proteins.

White skeletal muscle

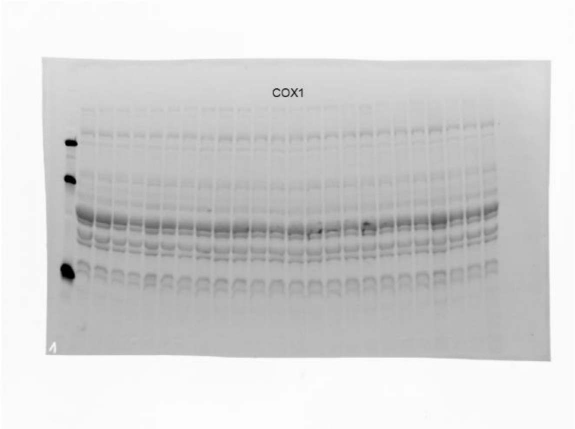

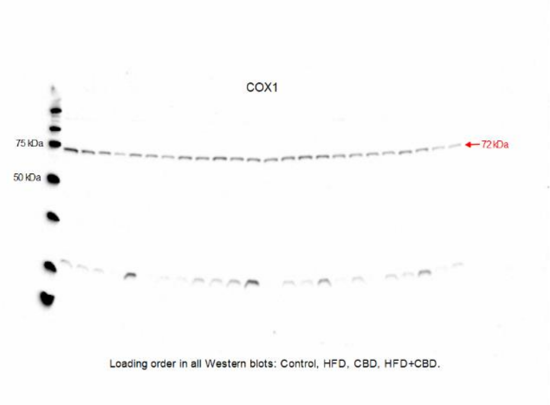

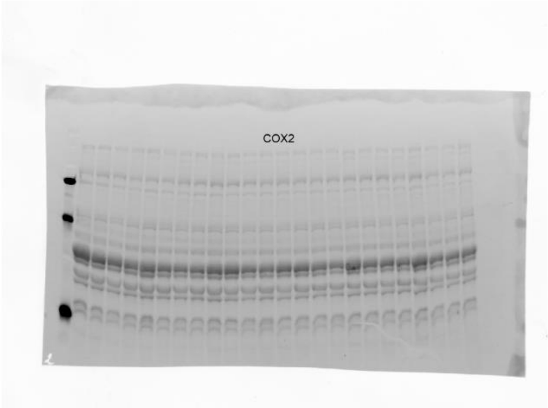

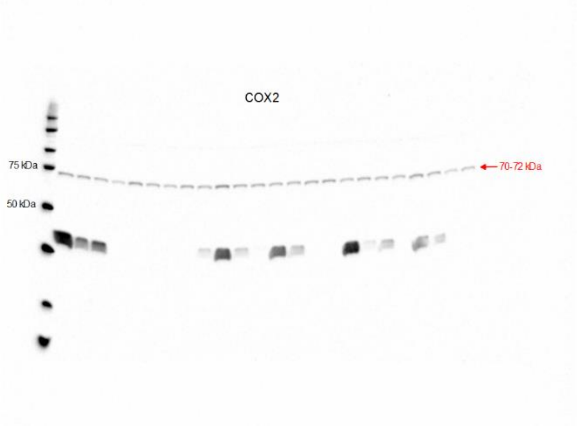

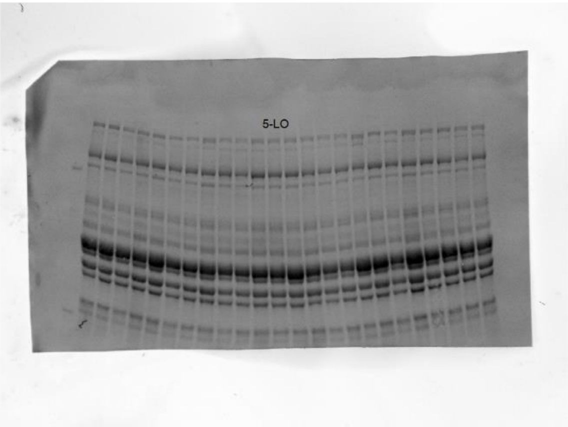
5-LO

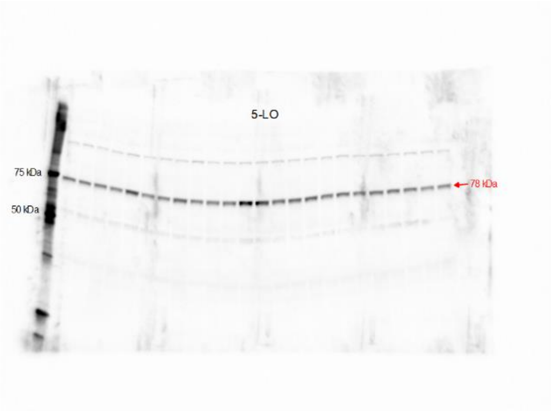
5-LO
75 kDa
50 kDa
78 kDa

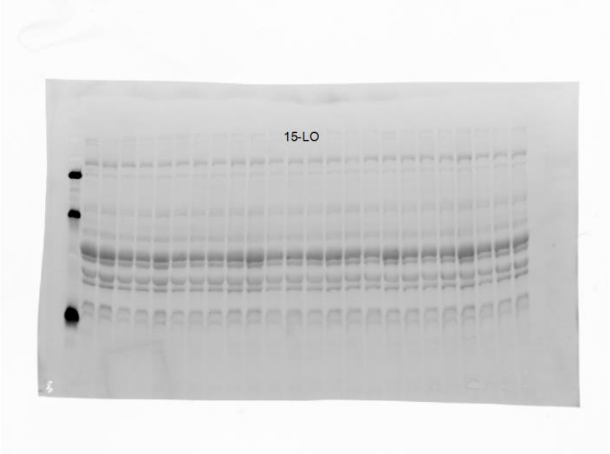
15-LO

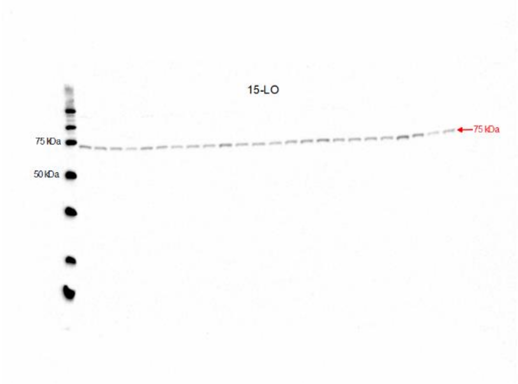
15-LO
75 kDa
50 kDa
75 kDa

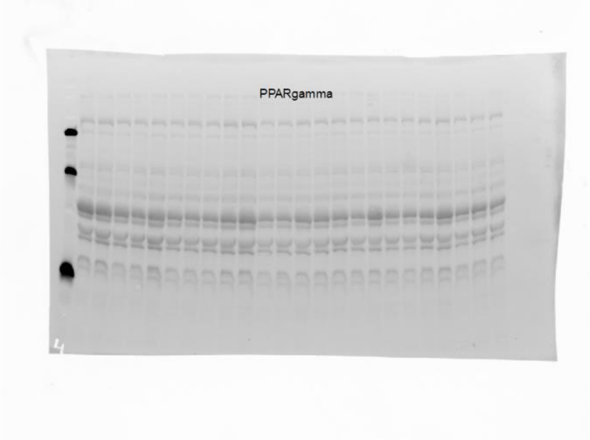
PPARgamma

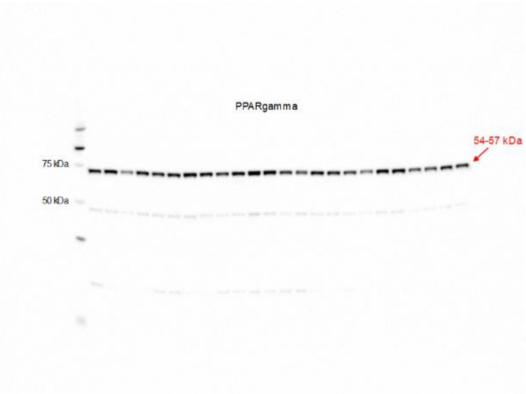
PPARgamma
54-57 kDa
75 kDa
50 kDa

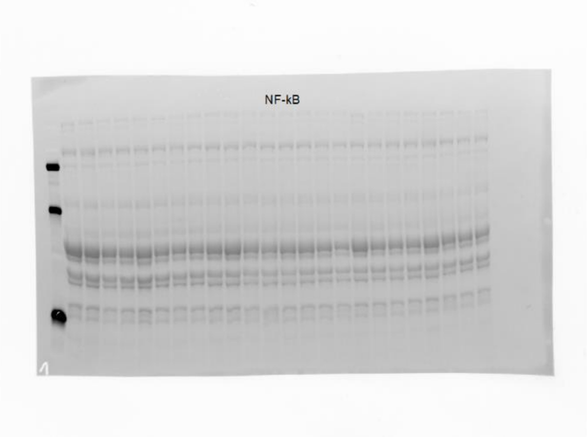
NF-kB

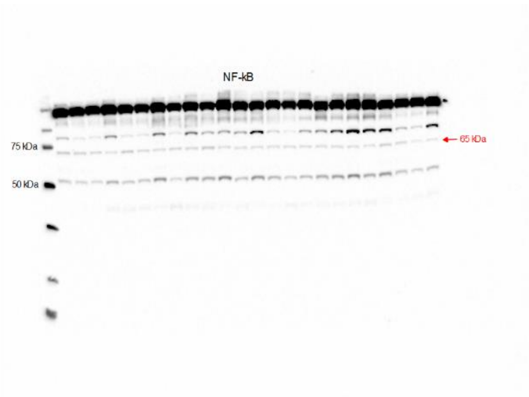
NF-kB
65 kDa
75 kDa
50 kDa

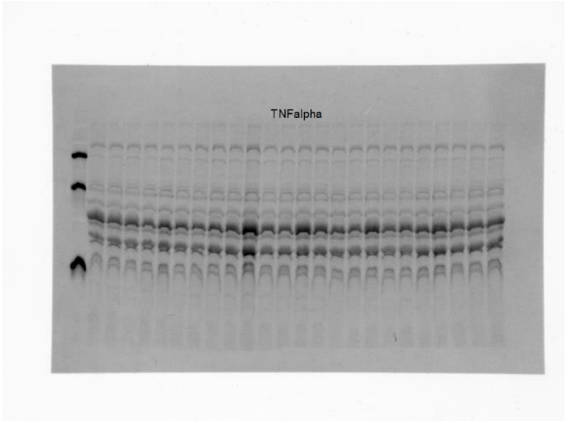
TNFalpha

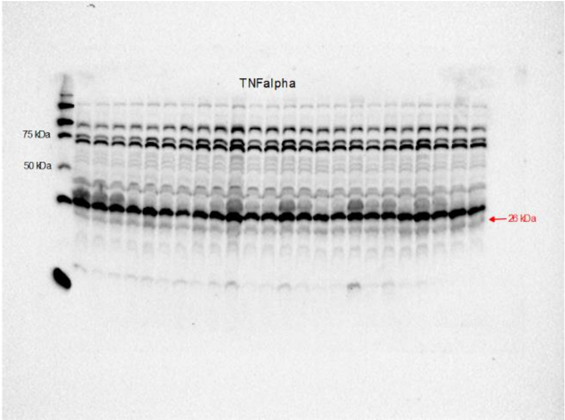
TNFalpha
75 kDa
50 kDa
28 kDa

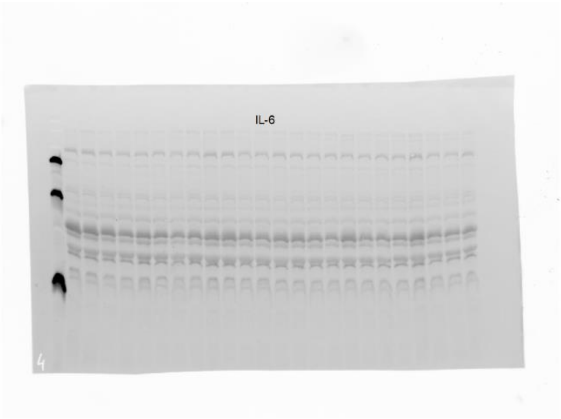
IL-6
4

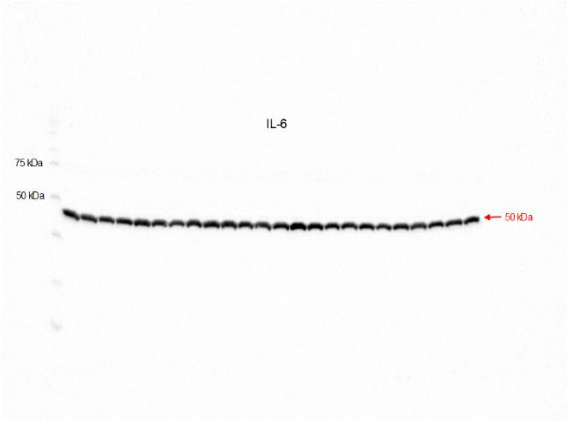
IL-6
75 kDa
50 kDa
50 kDa

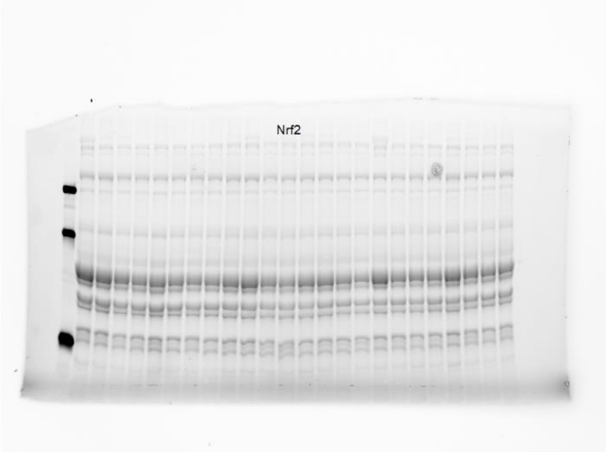
Nrf2

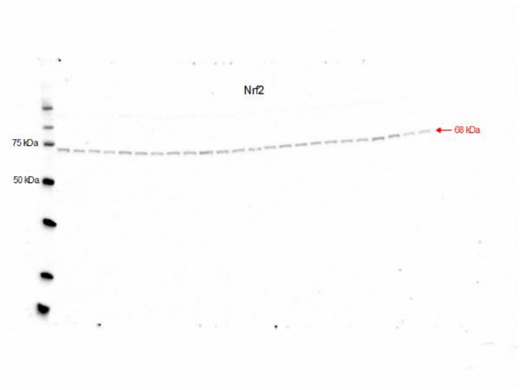
Nrf2
68 kDa
75 kDa
50 kDa

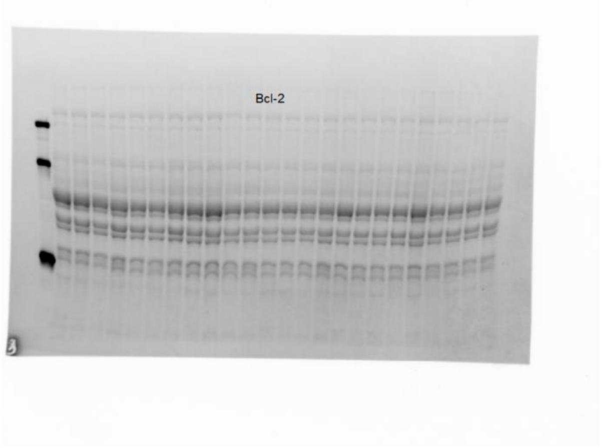
Bcl-2

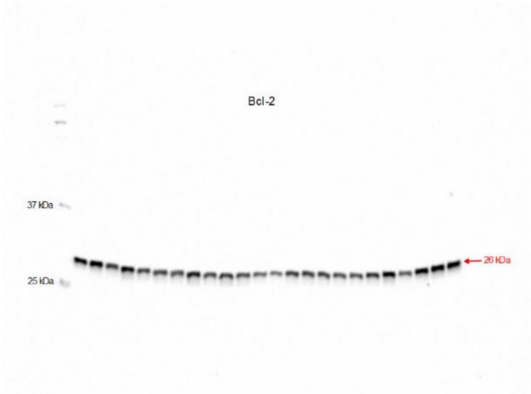
Bcl-2
37 kDa
26 kDa
25 kDa

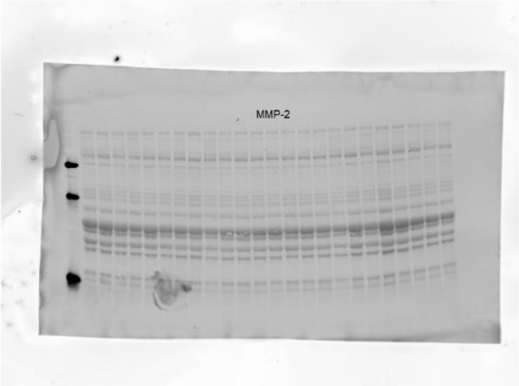
MMP-2

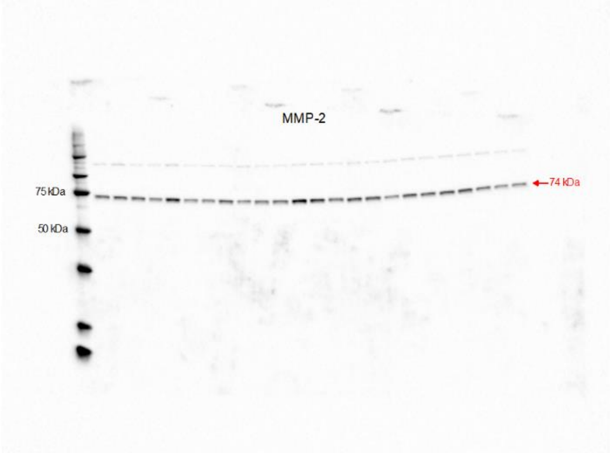
MMP-2
75 kDa
50 kDa
74 kDa

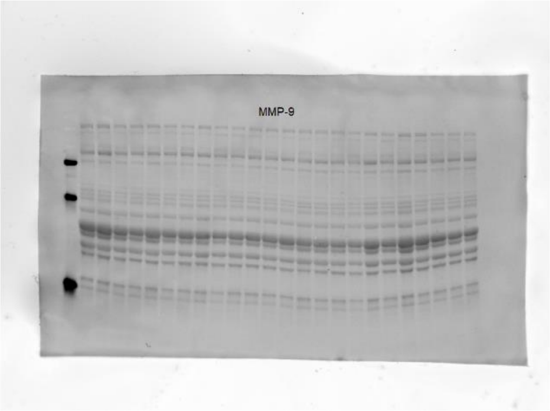
MMP-9

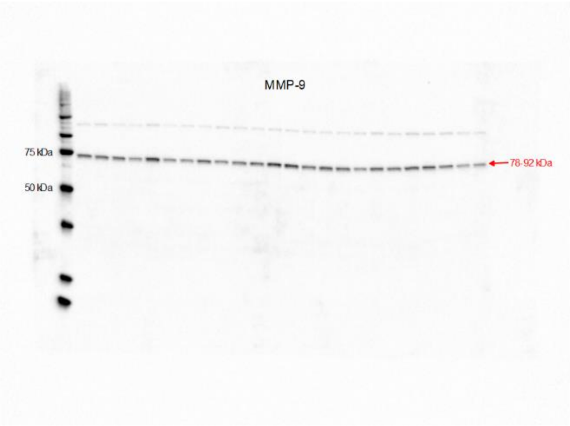
MMP-9
75 kDa
50 kDa
78-92 kDa

Red skeletal muscle

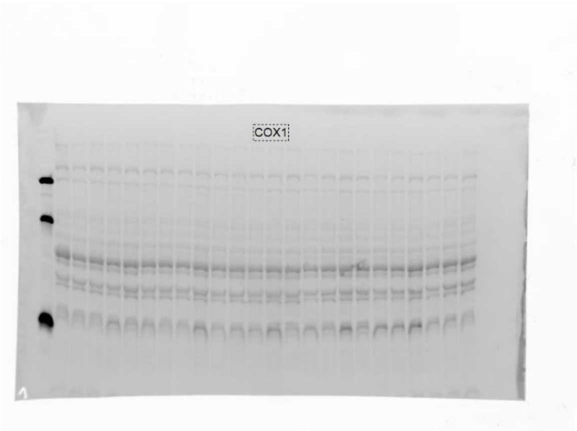

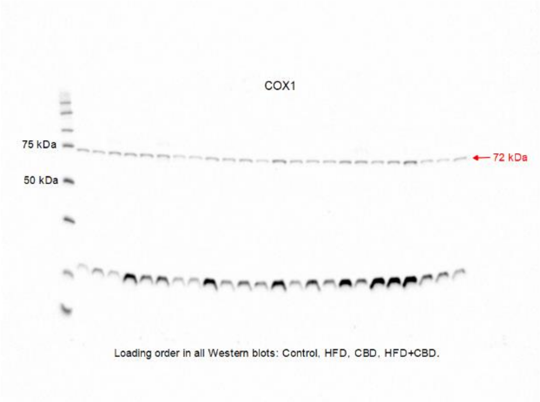

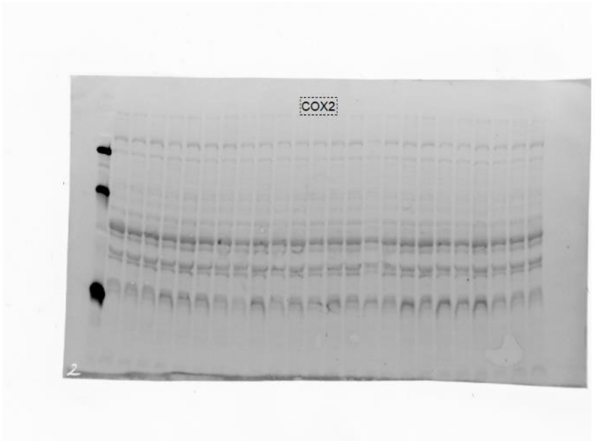

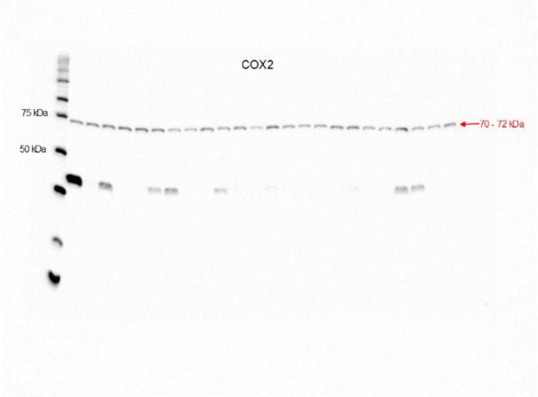

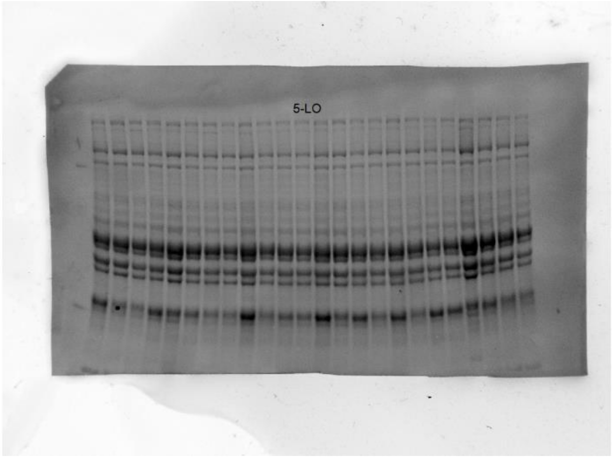
5-LO

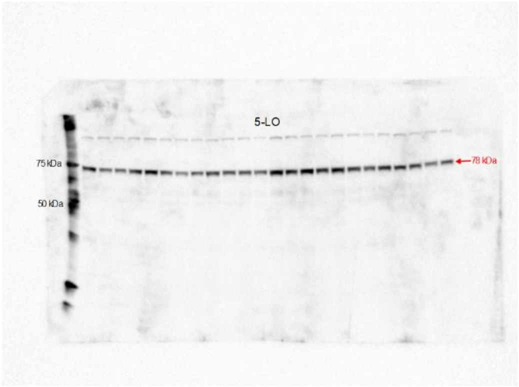
5-LO
75 kDa
50 kDa
78 kDa

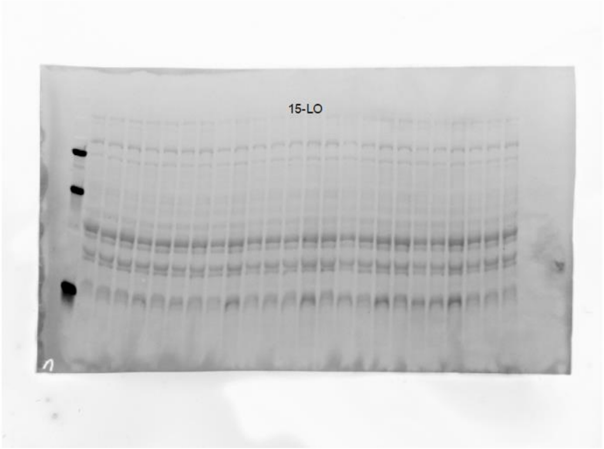
15-LO

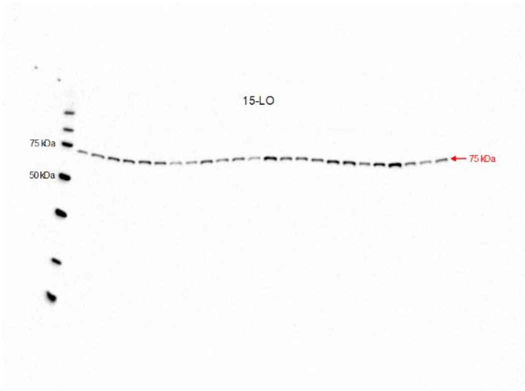
15-LO
75 kDa
50 kDa
75 kDa

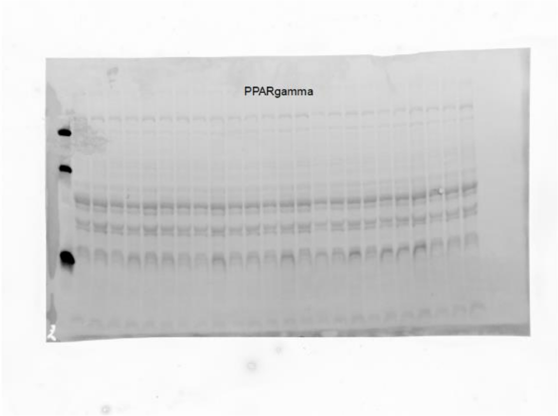
PPARgamma

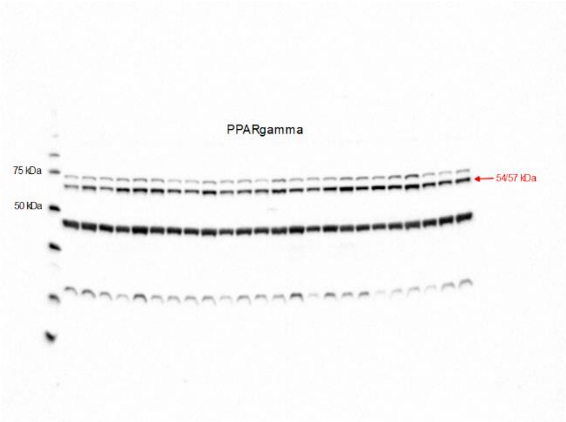
PPARgamma
75 kDa
50 kDa
54/57 kDa

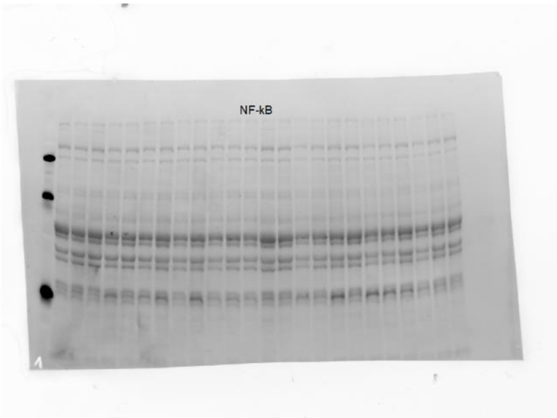
NF-kB

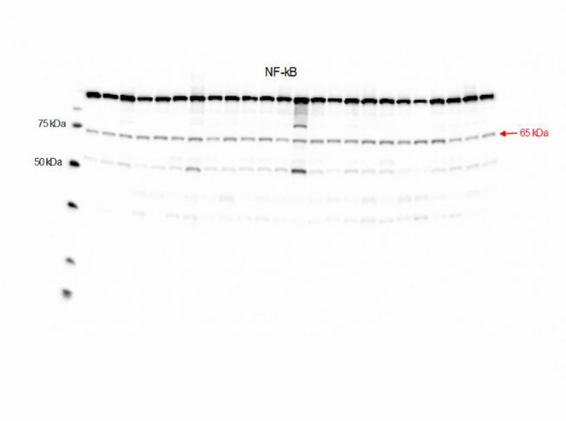
NF-kB
75 kDa
50 kDa
65 kDa

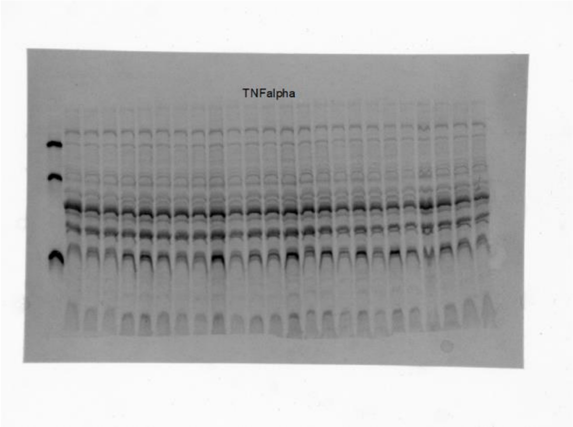
TNFalpha

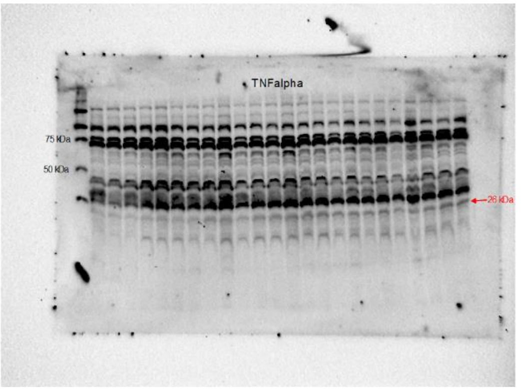
TNFalpha
75 kDa
50 kDa
26 kDa

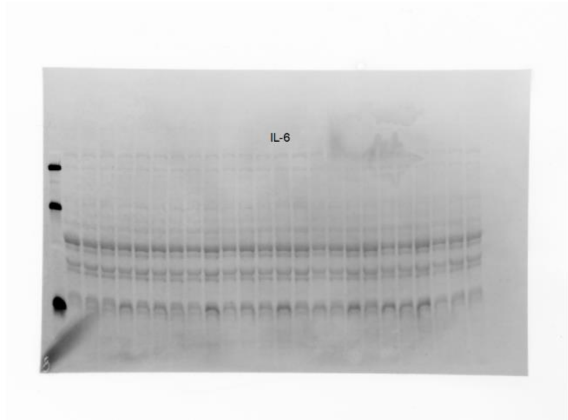
IL-6

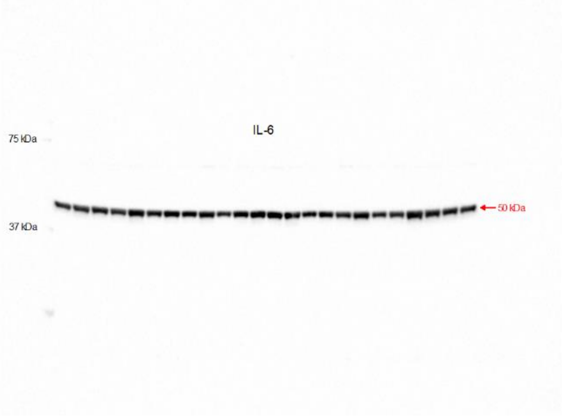
IL-6
75 kDa
50 kDa
37 kDa

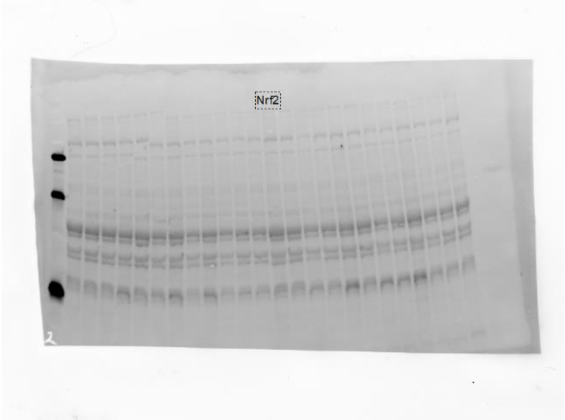
Nrf2

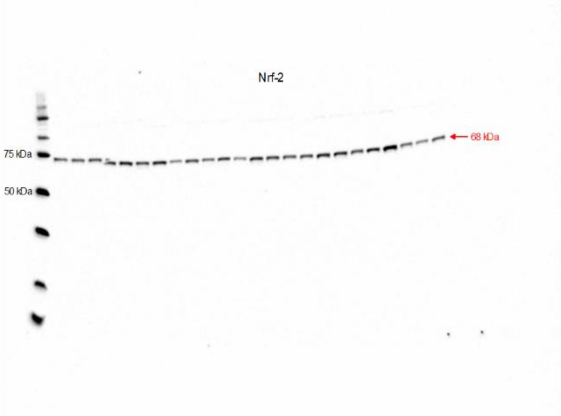
Nrf-2
75 kDa
50 kDa
68 kDa

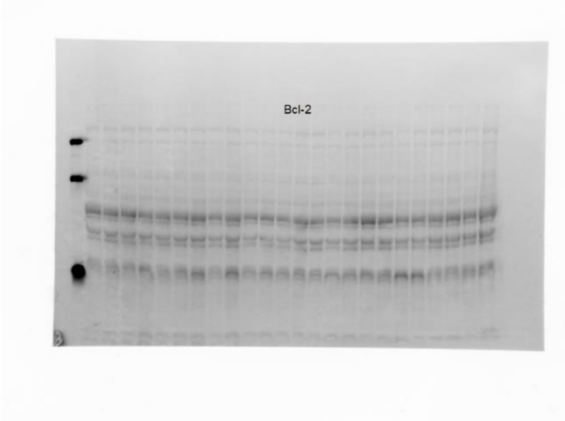
Bcl-2

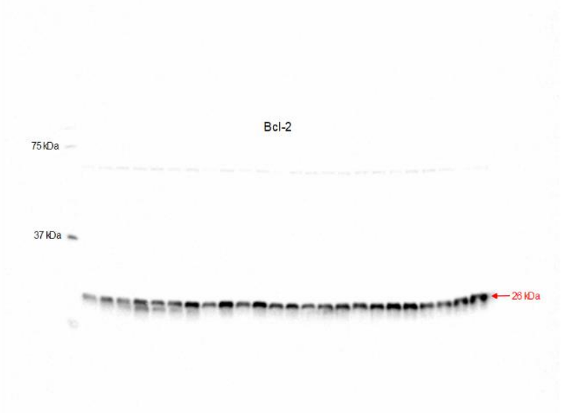
Bcl-2
75 kDa
37 kDa
26 kDa

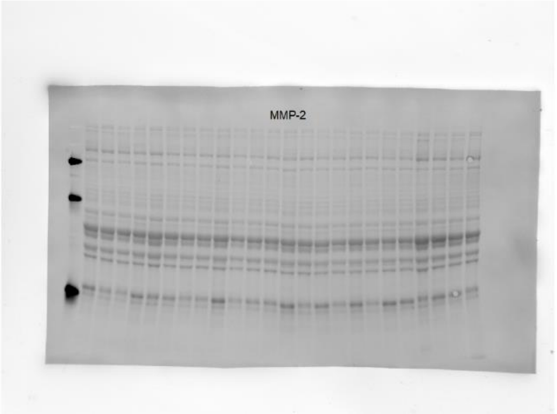
MMP-2

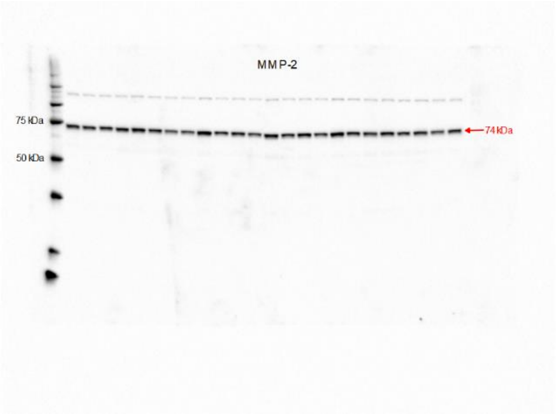
MMP-2
75 kDa
50 kDa
74 kDa

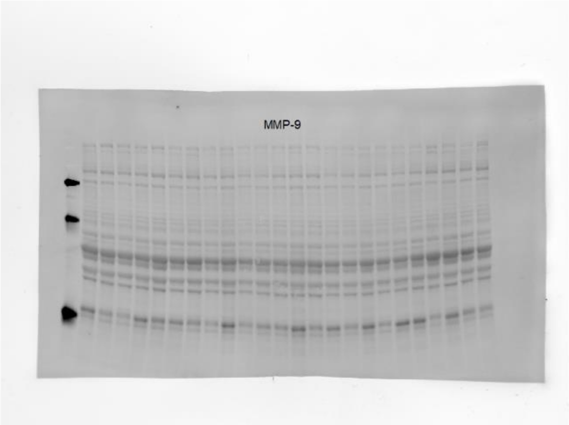
MMP-9

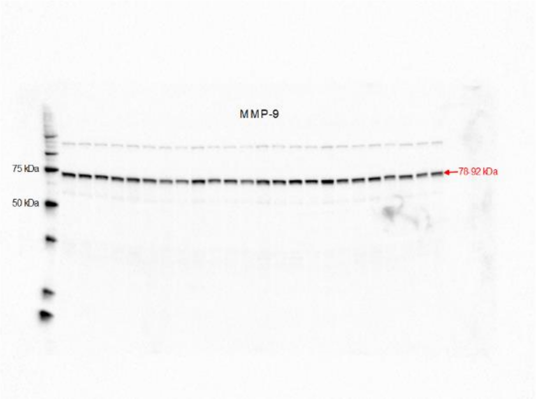
MMP-9
75 kDa
50 kDa
78.92 kDa
